# Confirmation of a Causal *Taar1* Allelic Variant in Addiction-Relevant Methamphetamine Behaviors

**DOI:** 10.3389/fpsyt.2021.725839

**Published:** 2021-08-26

**Authors:** Tamara J. Phillips, Tyler Roy, Sara J. Aldrich, Harue Baba, Jason Erk, John R. K. Mootz, Cheryl Reed, Elissa J. Chesler

**Affiliations:** ^1^Department of Behavioral Neuroscience and Methamphetamine Abuse Research Center, Oregon Health & Science University, Portland, OR, United States; ^2^Veterans Affairs Portland Health Care System, Portland, OR, United States; ^3^The Jackson Laboratory and Center for Systems Neurogenetics of Addiction, Bar Harbor, ME, United States

**Keywords:** aversion, CRISPR-*Cas9*, hypothermia, knock-in, morphine, pleiotropic, selective breeding, two-bottle choice

## Abstract

Sensitivity to rewarding and reinforcing drug effects has a critical role in initial use, but the role of initial aversive drug effects has received less attention. Methamphetamine effects on dopamine re-uptake and efflux are associated with its addiction potential. However, methamphetamine also serves as a substrate for the trace amine-associated receptor 1 (TAAR1). Growing evidence in animal models indicates that increasing TAAR1 function reduces drug self-administration and intake. We previously determined that a non-synonymous single nucleotide polymorphism (SNP) in *Taar1* predicts a conformational change in the receptor that has functional consequences. A *Taar1*^*m*1*J*^ mutant allele existing in DBA/2J mice expresses a non-functional receptor. In comparison to mice that possess one or more copies of the reference *Taar1* allele (*Taar1*^+/+^ or *Taar1*^+/*m*1*J*^), mice with the *Taar1*^*m*1*J*/*m*1*J*^ genotype readily consume methamphetamine, express low sensitivity to aversive effects of methamphetamine, and lack sensitivity to acute methamphetamine-induced hypothermia. We used three sets of knock-in and control mice in which one *Taar1* allele was exchanged with the alternative allele to determine if other methamphetamine-related traits and an opioid trait are impacted by the same *Taar1* SNP proven to affect MA consumption and hypothermia. First, we measured sensitivity to conditioned rewarding and aversive effects of methamphetamine to determine if an impact of the *Taar1* SNP on these traits could be proven. Next, we used multiple genetic backgrounds to study the consistency of *Taar1* allelic effects on methamphetamine intake and hypothermia. Finally, we studied morphine-induced hypothermia to confirm prior data suggesting that a gene in linkage disequilibrium with *Taar1*, rather than *Taar1*, accounts for prior observed differences in sensitivity. We found that a single SNP exchange reduced sensitivity to methamphetamine conditioned reward and increased sensitivity to conditioned aversion. Profound differences in methamphetamine intake and hypothermia consistently corresponded with genotype at the SNP location, with only slight variation in magnitude across genetic backgrounds. Morphine-induced hypothermia was not dependent on *Taar1* genotype. Thus, *Taar1* genotype and TAAR1 function impact multiple methamphetamine-related effects that likely predict the potential for methamphetamine use. These data support further investigation of their potential roles in risk for methamphetamine addiction and therapeutic development.

## Introduction

Considerable research has focused on drug use disorders as motivational disorders involving inherent or drug-induced reward pathway function. Human and animal research supports a critical role for circuitry underlying sensitivity to rewarding and reinforcing drug effects in risk for continued use, neuroadaptation and relapse. Less is known about factors that reduce risk for addiction such as, for example, the protective role of sensitivity to aversive or adverse drug effects. Although there is important research in this area for alcohol (1–4), it has not been a focus of psychostimulant research or therapeutic development for psychostimulant addiction. To address initial sensitivity to drug aversive effects in humans requires knowledge of initial drug effects and the inclusion of individuals in studies who have tried a drug, but did not continue to use because they found the effects to be unpleasant. This is not the typical research performed in the addiction field; rather individuals suffering from a substance use disorder, or with a significant history of drug use, are compared to individuals with a low to modest drug history. Concerns about potential long-term consequences on behavior and in the brain, of even relatively low-level exposure to drugs like methamphetamine (MA) (5–14), raise concerns about conducting such research in drug-naïve humans. However, the study of drug avoiders could lead to the identification of a new class of therapeutics. Animal models of drug use have an important role in this area of study, because drug history can be controlled and initial responses readily measured.

Bidirectional selective breeding has the explicit goal of creating animal lines that exhibit a low vs. high level of a particular characteristic. We bred mice for level of voluntary MA intake and created MA high drinking (MAHDR) and MA low drinking (MALDR) lines of mice that consume binge-like levels of MA or avoid consuming MA, respectively (15–18). Using this model, we determined that a single nucleotide polymorphism (SNP) at position 229 in the trace amine-associated receptor 1 gene (*Taar1*) accounts for 60% of the heritable variance in MA intake (19–21). The non-synonymous SNP (rs33645709) is found in DBA/2J mice sourced from The Jackson Laboratory (JAX), but not in DBA/2 mice sourced from other suppliers (22) or in any of the 28 other strains that have been genotyped at this genetic location (23, 24). This coding variant changes a proline to a threonine in the second transmembrane domain of the receptor (24). Multiple lines of evidence, beginning with quantitative trait locus mapping and culminating in the use of a CRISPR-*Cas9*-derived knock-in (KI) model on the MAHDR background, definitively determined that this *Taar1* SNP impacts level of MA consumption (21). Thus, mice homozygous for the *Taar1*^*m*1*J*^ allele consume more MA on average than mice that possess one or two copies of the reference *Taar1*^+^ allele.

In addition to identifying genetic differences related to the bidirectional selection response, selectively bred lines provide information about genetically correlated traits, defined as phenotypes that are impacted by one or more of the genes that influence the selection trait. Several MA-related traits reliably differentiate the MA drinking (MADR) lines. These include rewarding and aversive traits, as well as a physiological trait, MA-induced hypothermia, proposed to be among the aversive effects of MA that inhibit MA intake (20, 25, 26). Herein, we report studies performed in three sets of matched KI and control line mice in which one *Taar1* allele was exchanged with the alternative allele. First, MAHDR-*Taar1*^+/+^ KI and control mice were studied for tastant intake and preference, and sensitivity to two traits hypothesized to be pleiotropically influenced by *Taar1*: sensitivity to MA-induced conditioned place preference and conditioned taste aversion. Based on previous findings (16, 18, 21, 22, 27), we predicted that the KI and control mice would not differ in saccharin or quinine intake or preference, KI of the *Taar1*^+^ allele would decrease sensitivity to MA-induced conditioned place preference, and KI of the *Taar1*^+^ allele would increase sensitivity to MA-conditioned taste aversion. Next, KI mice generated on the MADR progenitor C57BL/6J and DBA/2J background strains were studied for MA intake and MA-induced hypothermia to examine replication of *Taar1* effects on different genetic backgrounds. These traits have already been confirmed to be impacted by *Taar1* in the MAHDR KI and control lines (21).

Finally, previous data in MADR mice suggest that a difference in sensitivity to morphine-induced hypothermia was not a pleiotropic effect of *Taar1*, but more likely due to a linked polymorphism (25). To confirm this, we examined this trait in the MAHDR-*Taar1*^+/+^ KI and control mice and predicted comparable morphine-induced hypothermia.

## Materials and Methods

### Subjects

Subjects were male and female MAHDR*-Taar1*^+/+^ KI, DBA/2J*-Taar1*^+/+^ KI, C57BL/6J-*Taar1*^*m*1*J*/*m*1*J*^ KI, and control lines matched to each KI line (MAHDR-*Taar1*^*m*1*J*/*m*1*J*^, DBA/2J-*Taar1*^*m*1*J*/*m*1*J*^, and C57BL/6J-*Taar1*^+/+^). The MAHDR KI mice were created at the Oregon Health & Science University Transgenic Mouse Models Shared Resource, utilizing CRISPR-*Cas9* technology to replace the *Taar1*^*m*1*J*^ allele with the reference *Taar1*^+^ allele; controls were derived from those mice in which the *Taar1*^*m*1*J*^ allele was not successfully excised and replaced; thus, they retained the *Taar1*^*m*1*J*/*m*1*J*^ genotype. Details can be found in Stafford et al. (21). The identical process was applied at JAX (Bar Harbor, ME, USA) to generate the same KI on a pure DBA/2J inbred strain background, or to replace the reference *Taar1*^+^ allele with the *Taar1*^*m*1*J*^ allele on a pure C57BL/6J inbred strain background.

Mice participating in the current studies were either born within the VA Portland Health Care System (VAPORHCS) Veterinary Medical Unit or within a breeding colony at JAX; location for each study is indicated in experiment descriptions. Breeders (pairs at VAPORHCS; harems at JAX) were maintained in standard acrylic plastic shoebox cages on corncob bedding (The Andersons, Maumee, Ohio; VAPORHCS) or pine shavings (Hancock Lumber, Bethel, Maine; JAX), with wire lids and filter tops. Breeding cages resided on Thoren racks under a standard 12:12 light:dark cycle, and mice were weaned at 21 ± 2 days of age into same-sex groups of 2–4 per cage. During breeding and experiments, mice were maintained in climate-controlled rooms under a standard 12:12 light:dark cycle with lights on at 0600 h, and free access to water (tap water at the VAPORHCS; filtered and acidified at JAX) and rodent block food (Purina 5001 or 5LOD PicoLab Rodent Diet; Animal Specialties, Woodburn, Oregon at the VAPORHCS; NIH315K52 chow Lab Diet 6%PM Nutrition, St. Louis MO, USA at JAX); exceptions are noted in experimental methods. All animal care and testing procedures were approved by the VAPORHCS or JAX Institutional Animal Care and Use Committee, and were conducted in compliance with the National Institutes of Health Guidelines for Care and Use of Laboratory Animals. Group sizes for each experiment are given in the figure captions.

### Genotyping

All KI and control offspring used in these studies were genotyped for *Taar1* using an rtPCR method developed in our laboratory for the relevant *Taar1* SNP, based on a standard Taqman (ThermoFisher Scientific) assay in which fluorescent probes were used for differentiation (22).

### Drugs

(+)Methamphetamine hydrochloride was purchased from Sigma-Aldrich (St. Louis, MO, USA) or obtained from the National Institutes of Health, National Institute on Drug Abuse drug supply program (Rockville, MD, USA) and dissolved in tap water for drinking or in sterile 0.9% saline (Baxter Healthcare Corp., Deerfield, IL, USA) for injection. Sodium chloride for conditioned taste aversion studies, and saccharin and quinine for novel tastant intake studies, were purchased from Sigma-Aldrich and dissolved in tap water. Morphine was obtained from the NIDA Drug Supply program (Rockville, MD, USA) and dissolved in sterile saline. Saline vehicle served as a 0 dose control treatment for MA and morphine injection studies. All injections were delivered intraperitoneally (IP) in a volume of 10 ml/kg.

### Experiment 1: Novel Tastant Intake and Preference in MAHDR-*Taar1^+/+^* KI and MAHDR-*Taar1^*m*1*J*/*m*1*J*^* Control Mice

We characterized saccharin (SACC) and quinine (QUIN) intake and preference to investigate whether a difference in tastant intake or preference corresponds with the difference in MA intake found in a previous study between the MAHDR-*Taar1*^+/+^ KI and MAHDR-*Taar1*^*m*1*J*/*m*1*J*^ control mice (21). This study was conducted at the VAPORHCS using methods consistent with our previous studies in the MADR mice (16, 18). Male and female mice were weighed, singly-housed, and given access to two water-filled 25-ml graduated cylinders fitted with stoppers and sipper tubes for 2 consecutive days to familiarize them with drinking fluid from these tubes. Using a counterbalanced design, mice were then offered water vs. SACC and then water vs. QUIN or the two tastants in the alternate order. Tastants were offered 24 h/day in two increasing concentrations for 4 days each and mice were weighed every 4 days; therefore, the two-bottle choice tastant phase included days 3–18. The positions of the water and tastant tubes were alternated every 2 days, the SACC concentrations were 1.6 and 3.2 mM, and the QUIN concentrations were 0.015 and 0.03 mM, consistent with our previous studies (16, 18). Consumption was measured each day in ml. Mice were tested at an average age of 81 ± 1 days, with a range of 77–87 days.

### Experiment 2: MA-Induced Conditioned Place Preference Testing in MAHDR-*Taar1^+/+^* KI and MAHDR-*Taar1^*m*1*J*/*m*1*J*^* Control Mice

Group housed (2–4 per cage) male and female MAHDR-*Taar1*^+/+^ KI and MAHDR-*Taar1*^*m*1*J*/*m*1*J*^ control mice were tested for sensitivity to conditioned place preference induced by 0.5 mg/kg MA, a dose that has produced consistent preference in MAHDR and no preference in MALDR mice (16, 18). This study was conducted at the VAPORHCS using our established unbiased place conditioning procedure. Custom-built conditioning boxes (30 cm × 15 cm × 15 cm; San Diego Instruments, San Diego, CA USA) were housed in sound attenuating, illuminated and ventilated chambers. Each conditioning box had a removable central black wall used to sequester the animal to the left or right half of the chamber during conditioning trials; the wall was removed during preference tests. Removable floor panels with unique textures served as conditioning cues. One floor was composed of 2.3 mm stainless steel rods mounted 6.4 mm apart (the “grid” floor); the other was a stainless steel sheet with 6.4 mm round holes on 9.5 mm staggered centers (the “hole” floor). Animal location was detected by photocell beam interruptions and automatically converted to time spent on a particular floor type; photocell beam interruptions were also recorded as a measure of locomotor activity.

The study began on a Monday and excluded weekends, and mice were returned to group housing (2–4 per cage) each day after testing. Mice were moved to the procedure room each day 1 h before conditioning or testing. Initial preference for the cues was determined in a 30-min test; mice were injected with saline immediately prior to placement in the box. Beginning 24 h later, there were 12 alternating conditioning trials (one daily), six immediately after saline, and six immediately after 0.5 mg/kg MA treatment that were 15 min in duration. Half of the mice had MA paired with the grid floor and half with the hole floor, with left/right placement of floor types counterbalanced. A 30-min “drug-free” preference test was performed the day after the last conditioning trial; mice were treated with saline immediately prior to placement in the box. Finally, a 30-min “drug-present” preference test was performed 2 days after the drug-free preference test (after a weekend break); mice were treated with MA immediately prior to placement in the box. Mice were tested at an average age of 91 ± 1 days, with a range of 73–107 days; mice were tested between 1000 and 1400 h.

### Experiment 3: MA-Induced Conditioned Taste Aversion Testing in MAHDR-*Taar1^+/+^* KI and MAHDR-*Taar1^*m*1*J*/*m*1*J*^* Control Mice

Male and female MAHDR*-Taar1*^+/+^ KI and MAHDR-*Taar1*^*m*1*J*/*m*1*J*^ control mice were tested for sensitivity to conditioned taste aversion induced by 2 mg/kg MA, a dose that has produced consistent differences in mice with different *Taar1* genotypes (22, 27). This study was conducted at the VAPORHCS using our established procedures. A novel 0.2 M NaCl solution was offered as the conditioned cue just prior to MA treatment to create an association with the interoceptive effects of MA. Briefly, mice were weighed, singly-housed, and familiarized to drinking water from a 10-ml graduated cylinder fitted with a sipper tube (study days −1 and 0). Water access was then limited to 2 h per day for a 4-day acclimation period to induce motivation to drink the novel NaCl solution at a particular time of each day (study days 1–4). Beginning on day 5, the NaCl solution was offered for 1 h every other day for 6 presentations (days 5, 7, 9, 11, 13, and 15). No treatment was given after the first presentation, which was a trial intended to reduce neophobia. On the remaining NaCl access days, with the exception of the last, saline or MA was injected immediately after the drinking period. NaCl consumption was measured in ml. To ensure proper hydration, 3 h post-injection, mice were given access to water for 30 min, and they had 2 h water access on days between trials. Mice were tested at an average age of 93 ± 1 days, with a range of 82–112 days; NaCl access occurred at 0900–1000 h.

### Experiments 4 and 5: Two-Bottle Choice Methamphetamine Intake in DBA/2J*-Taar1^+/+^* KI and DBA/2J*-Taar1^*m*1*J*/*m*1*J*^* Control, and in C57BL/6J-*Taar1^*m*1*J*/*m*1*J*^* KI and C57BL/6J-*Taar1^+/+^* Control Mice

A two-bottle choice MA vs. water drinking procedure, similar to that used to characterize voluntary MA intake in our previous studies was used (15–18). This study was conducted at JAX. One day prior to testing, male and female mice were singly housed and offered two water-filled 50 ml polypropylene centrifuge tubes (item number 430291; Corning, Corning, NY) fitted with rubber stoppers (Fisher Scientific, Pittsburgh, PA, USA) and single ball-bearing stainless steel sipper tubes (Sta Pure Systems, Carnegie, PA, USA) to provide experience with consuming fluid from these tubes. On test days 2–5, mice were offered one water tube and a tube containing a 10 mg/L solution of MA in water. On days 6–17, the tubes contained water vs. 20 mg/L, then 40 mg/L, and then 80 mg/L MA, with each MA concentration offered for 4 consecutive days. During the MA phase, mice had access to MA 24 h/day and tubes were weighed prior to cage placement and again every 48 h. Changes in MA concentration were accompanied by fresh tubes and switching of the position of the water vs. MA tube to account for potential side bias in fluid intake. Mice were weighed on days 1 and 17 of the study, and weights on those days were averaged to approximate mg/kg MA consumed. Two separate studies were conducted. The first included DBA/2J*-Taar1*^+/+^ KI and DBA/2J*-Taar1*^*m*1*J*/*m*1*J*^ control mice, tested at an average age of 72 ± 2 days, with a range of 56–88 days. The other included C57BL/6J-*Taar1*^*m*1*J*/*m*1*J*^ KI and C57BL/6J-*Taar1*^+/+^ control mice, tested at an average age of 75 ± 1 days, with a range of 55–88 days.

### Experiments 6 and 7: MA-Induced Body Temperature Changes in DBA/2J*-Taar1^+/+^* and DBA/2J*-Taar1^*m*1*J*/*m*1*J*^* Control, and in C57BL/6J-*Taar1^*m*1*J*/*m*1*J*^* KI and C57BL/6J-*Taar1^+/+^* Control Mice

Male and female mice were tested for the effect of 2 mg/kg MA on core body temperature using our established procedures. This MA dose consistently produces hypothermia in MALDR mice and other mice that possess *Taar1*^+^, a response that is absent in MAHDR mice and other mice that lack TAAR1 function (20, 22). This study was conducted at the VAPORHCS using our established procedures. Mice were moved to the procedure room at 0800–0830 h, weighed, isolated in acrylic plastic cubicles to prevent huddling-associated body temperature changes, and left undisturbed for 1 h to acclimate to the testing environment, maintained at a temperature of 21 ± 1°C. A baseline temperature was then obtained at 0900–0930 h, designated as time 0 (T0), using a 5 mm glycerin-coated rectal probe attached to a Thermalert TH-8 digital thermometer (Sensortek, Clifton, New Jersey). Mice were then immediately treated with saline or 2 mg/kg MA and returned to their holding cubicles. Temperatures were subsequently obtained at T30, T60, T90, T120, T150, and T180 min post-injection. Experiment 6 included DBA/2J*-Taar1*^+/+^ KI and DBA/2J*-Taar1*^*m*1*J*/*m*1*J*^ control mice, tested at an average age of 92 ± 1 days, with a range of 62–122 days. Experiment 7 included C57BL/6J-*Taar1*^*m*1*J*/*m*1*J*^ KI and C57BL/6J-*Taar1*^+/+^ control mice, tested at an average age of 82 ± 1 days, with a range of 62–108 days.

### Experiment 8: Morphine-Induced Body Temperature Changes in MAHDR-*Taar1^+/+^* KI and MAHDR-*Taar1^*m*1*J*/*m*1*J*^* Control Mice

Male and female MAHDR-*Taar1*^+/+^ KI and MAHDR-*Taar1*^*m*1*J*/*m*1*J*^ control mice were tested for sensitivity to the hypothermic effect of 15 and 30 mg/kg morphine. These doses were chosen from our previous research in the MADR lines (25). This study was conducted at the VAPORHCS, and experimental details were identical to those described for experiments 6 and 7. Mice were tested at an average age of 85 ± 1 days, with a range of 65–109 days.

### Statistics

Data were analyzed using factorial ANOVA with repeated measures as appropriate. Independent grouping factors are described with the results for each study. Complex interactions involving more than 2 factors were first examined by 2-way ANOVA at each level of the third factor. Significant 2-way interactions were examined for simple main effects and means were compared using the Newman–Keuls *post-hoc* test. The number of *post-hoc* comparisons was reduced by assessing changes from one mean to the next for concentration, time, and trial effects, or between first and subsequent trials.

## Results

### Baseline Data

Baseline body weight data collected across studies, along with age ranges, are summarized in Table 1. Although there were some significant differences between genotypes in baseline body weight, differences were not consistently found across studies, suggesting that they were specific to the particular groups of animals included in a given experiment. Thus, C57BL/6J-*Taar1*^*m*1*J*/*m*1*J*^ KI and C57BL/6J-*Taar1*^+/+^ control mice had equivalent body weights in both studies in which they were tested; DBA/2J*-Taar1*^+/+^ KI and DBA/2J-*Taar1*^*m*1*J*/*m*1*J*^ control mice differed in body weight in one, but not the other experiment; and MAHDR-*Taar1*^+/+^ KI and MAHDR-*Taar1*^*m*1*J*/*m*1*J*^ control mice had comparable body weights in two studies and differed in the remaining two. When significant, mean differences ranged from 1.1 to 2.2 g. Total volume of water consumed was recorded prior to tastant access in Experiment 1 and there was no significant difference between the genotypes (mean ± SEM = 5.8 ± 0.2 ml vs. 6.0 ± 0.2 ml for KI and Control, respectively). Other measures, including total volume of fluid consumed, baseline locomotor activity level, and baseline body temperature are reported with the experiments during which these data were collected.

**Table 1:** Body weight and age range data for each study.

				**Body weight (g** **±** **SEM)**		
**Exp number**	**Study description**	**Mouse model**	**Age range (days)**	**KI**	**Control**	**Mouse line comparison**
(1)	Tastant intake	MAHDR-*Taar1^+/+^* KI vs. Control	77–87	25.1 ± 0.7	25.7 ± 0.7	KI = Control
(2)	MA conditioned place preference	MAHDR-*Taar1^+/+^* KI vs. Control	73–107	25.7 ±0.4	27.9 ± 0.4^***^	KI < Control
(3)	MA conditioned taste aversion	MAHDR-*Taar1^+/+^* KI vs. Control	82–112	26.8 ± 0.6	27.9 ± 0.6	KI = Control
(4)	MA intake	DBA/2J-*Taar1^+/+^* KI vs. Control	56–88	21.2 ± 0.7	20.5 ± 0.7	KI = Control
(5)	MA intake	C57BL/6J-*Taar1^*m*1*J*/*m*1*J*^* KI vs. Control	55–88	23.0 ± 0.5	23.8 ± 0.3	KI = Control
(6)	MA body temperature	DBA/2J-*Taar1^+/+^* KI vs. Control	62–122	25.2 ± 0.4	27.0 ± 0.4^**^	KI < Control
(7)	MA body temperature	C57BL/6J-*Taar1^*m*1*J*/*m*1*J*^* KI vs. Control	62–108	24.0 ± 0.2	23.9 ± 0.2	KI = Control
(8)	Morphine body temperature	MAHDR-*Taar1^+/+^* KI vs. Control	65–109	26.1 ± 0.3	27.1 ± 0.3^*^	KI < Control

*Exp, experiment; g, gram; KI, knock-in; MA, methamphetamine; MAHDR, methamphetamine high drinking mice; SEM, standard error of the mean; Taar1^+/+^, homozygous reference trace amine-associated receptor 1 genotype; Taar1^m1J/m1J^, homozygous mutant trace amine-associated receptor 1 genotype;*

* *p < 0.05*,

** *p < 0.01*,

*** *p < 0.001 for the difference between mouse lines*.

### Experiment 1: Novel Tastant Intake and Preference in MAHDR-*Taar1^+/+^* KI and MAHDR-*Taar1^*m*1*J*/*m*1*J*^* Control Mice

Data for each tastant were analyzed separately by repeated measures factorial ANOVA grouped on mouse line and sex, with concentration as the repeated measure.

#### SACC Intake, Preference, and Total Volume Consumed

**Figure 1:**
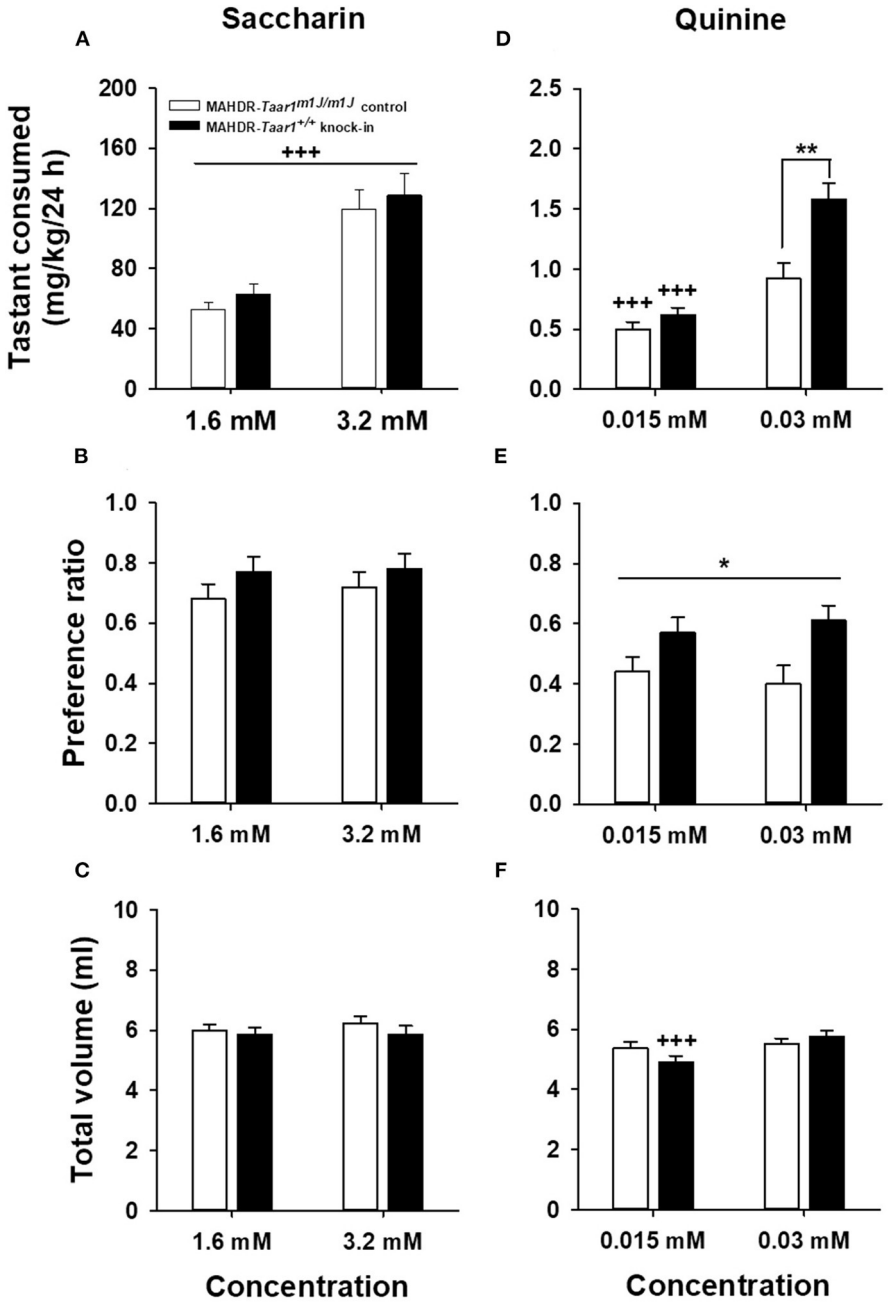
Novel saccharin and quinine tastant intake and preference in MAHDR-*Taar1*^+/+^ KI and MAHDR-*Taar1*^*m*1*J*/*m*1*J*^ control mice. Shown are means ± SEM for **(A)** saccharin consumed (mg/kg/24 h), **(B)** saccharin preference ratio (ml from saccharin tube/total ml consumed), **(C)** total volume (ml/24 h) consumed (water + saccharin solution) during access to each saccharin concentration, **(D)** quinine consumed (mg/kg 24 h), **(E)** quinine preference ratio (ml from quinine tube/total ml consumed), and **(F)** total volume consumed (water + quinine solution) during quinine access. Tastants were offered vs. water for 4-day periods at increasing concentrations in counterbalanced order. Total *N* = 48 mice (12 mice per sex for the MAHDR-*Taar1*^+/+^ KI; 10 female MAHDR-*Taar1*^*m*1*J*/*m*1*J*^ control mice, and 14 male MAHDR-*Taar1*^*m*1*J*/*m*1*J*^ control mice). *p < 0.05, **p < 0.01 for the main effect of mouse line **(E)** or for the line difference at the indicated concentration **(D)**; ^+++^*p* < 0.001 for the main effect of concentration **(A)** or for the concentration difference for the indicated mouse line **(D,F)**. MAHDR, methamphetamine high drinking mice; *Taar1*^+/+^, homozygous reference trace amine-associated receptor 1 genotype; *Taar1*^*m*1*J*/*m*1*J*^, homozygous mutant trace amine-associated receptor 1 genotype.

MAHDR-*Taar1*^+/+^ KI and MAHDR-*Taar1*^*m*1*J*/*m*1*J*^ control mice did not differ in SACC intake or preference (calculated as ml from tastant tube/total ml consumed). The initial analysis of SACC intake data (Figure 1A) identified significant effects of sex [*F*_(1, 44)_ = 4.7, *p* = 0.036] and SACC concentration [*F*_(1, 44)_ = 124.2, *p* < 0.0001]. Female mice consumed more SACC than males (mean ± SEM = 106.6 ± 10.4 mg/kg vs. 77.9 ± 6.6 mg/kg, respectively), and mice consumed more SACC when it was offered at the higher concentration. These outcomes were not dependent on mouse line. For SACC preference (Figure 1B) and total volume consumed from the water plus SACC tubes (Figure 1C), there were no significant effects of line, sex, or concentration.

#### QUIN Intake, Preference, and Total Volume Consumed

MAHDR-*Taar1*^+/+^ KI mice consumed more QUIN and had a higher QUIN preference ratio, compared to MAHDR-*Taar1*^*m*1*J*/*m*1*J*^ control mice. The initial analysis of QUIN intake data (Figure 1D) identified a significant line x QUIN concentration interaction [*F*_(1, 44)_ = 13.9, *p* = 0.0005]. There were no effects of sex. Follow-up analyses indicated that more QUIN was consumed by mice of both lines when the concentration was increased. The lines consumed comparable amounts of QUIN at the lower concentration, but MAHDR-*Taar1*^+/+^ KI mice consumed more QUIN than MAHDR-*Taar1*^*m*1*J*/*m*1*J*^ control mice when the QUIN concentration was increased (*p* = 0.001). For QUIN preference (Figure 1E), there was a significant main effect of line [*F*_(1, 44)_ = 6.2, *p* = 0.02], with MAHDR-*Taar1*^+/+^ KI mice exhibiting higher preference than MAHDR-*Taar1*^*m*1*J*/*m*1*J*^ control mice. For total volume consumed (Figure 1F), there was a significant line x concentration interaction [*F*_(1, 44)_ = 11.6, *p* = 0.001] that was associated with smaller volumes consumed by MAHDR-*Taar1*^+/+^ KI mice when the lower vs. higher QUIN concentration was offered (*p* < 0.0001; 4.9 ± 0.2 vs. 5.8 ± 0.2 for the 0.015 and 0.03 mM concentrations, respectively). However, there were no significant differences between the lines in total volume consumed at either concentration.

### Experiment 2: MA-Induced Conditioned Place Preference Testing in MAHDR-*Taar1^+/+^* KI and MAHDR-*Taar1^*m*1*J*/*m*1*J*^* Control Mice

**Figure 2:**
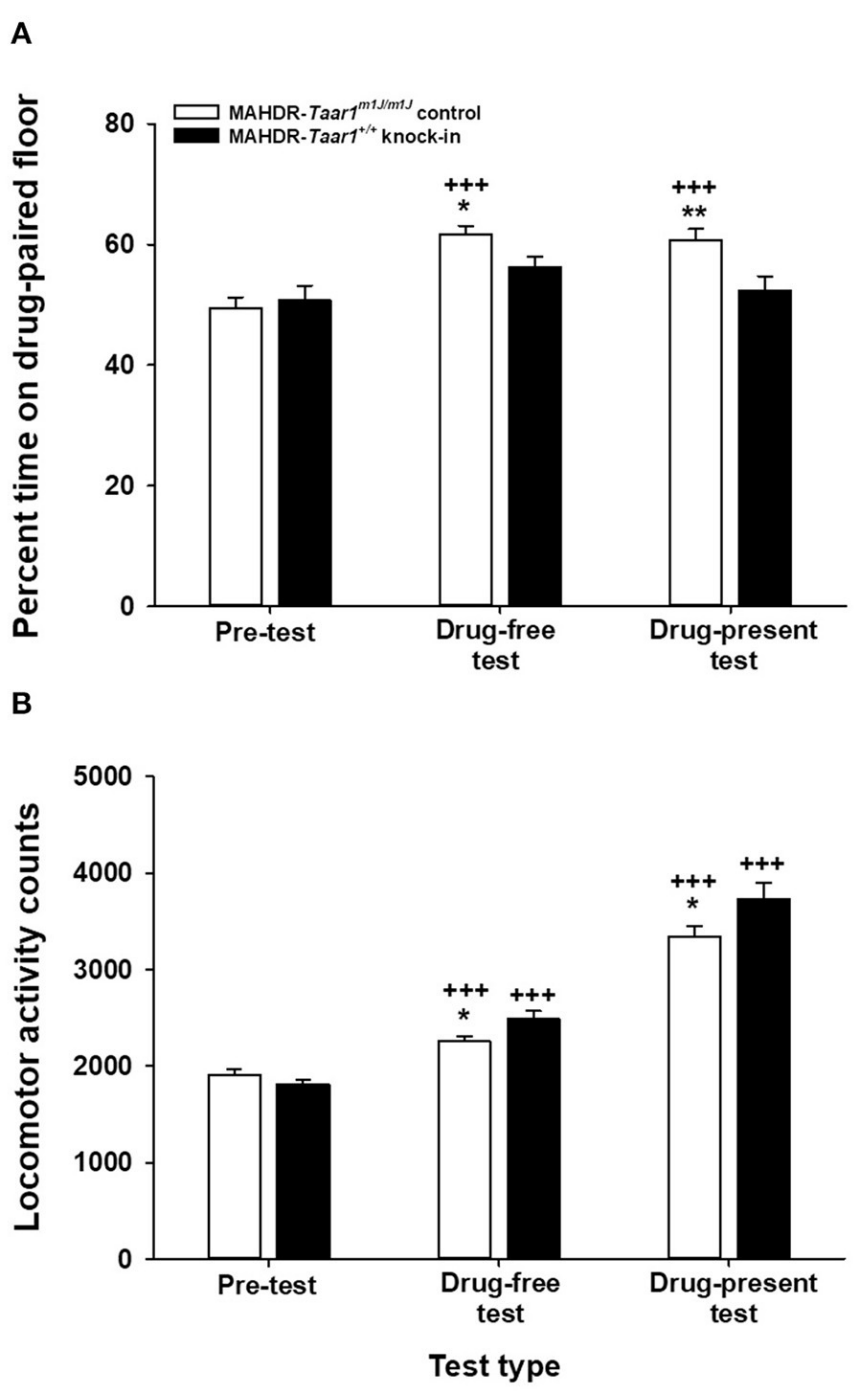
MAHDR-*Taar1*^*m*1*J*/*m*1*J*^ control mice, but not MAHDR-*Taar1*^+/+^ KI mice, exhibit MA-induced conditioned place preference and are less active during preference testing. Shown are means ± SEM for **(A)** percent time on drug-paired floor during the pre-test, drug-free test, and drug-present test and for **(B)** locomotor activity counts during the three tests. Mice were treated with saline prior to preference testing for the pre-test and drug-free test, and with 0.5 mg/kg MA prior to the drug-present test; tests were 30 min in duration. Total *N* = 96 mice (12 mice per line per sex were conditioned with MA on the grid floor; 12 mice per line per sex were conditioned with MA on the hole floor). *p ≤ 0.05, **p < 0.01 for the line difference; ^+++^*p* < 0.001 for the difference from pre-test in **(A)** and for the difference from pre-test and drug-free or drug-present test in **(B)**. MA, methamphetamine; MAHDR, methamphetamine high drinking mice; *Taar1*^+/+^, homozygous reference trace amine-associated receptor 1 genotype; *Taar1*^*m*1*J*/*m*1*J*^, homozygous mutant trace amine-associated receptor 1 genotype.

Data analyses considered percent time spent on the drug-paired floor during the pre-test, drug-free test and drug-present test, as measures of initial floor type bias, preference for floor cues induced by prior association with MA, and preference for MA-associated floor cues when tested during the associative state (Figure 2A). Locomotor activity data collected during these tests were also analyzed (Figure 2B). Data were analyzed by repeated measures factorial ANOVA grouped on mouse line and sex, with test day as the repeated measure.

#### Place Preference

Floor cues were initially equally preferred, and MA induced a conditioned preference in MAHDR-*Taar1*^*m*1*J*/*m*1*J*^ control mice, but not MAHDR-*Taar1*^+/+^ KI mice. There was a significant line × test day interaction [*F*_(2, 184)_ = 3.54, *p* = 0.03], but no significant effects of sex. For initial preference, there was no significant difference between the lines for percent time spent on the assigned drug-paired floor, and values were near 50%, indicating that the floor types were approximately equally preferred before conditioning. For the drug-free preference test, there was a significant difference between the lines (*p* = 0.02), with the MAHDR-*Taar1*^*m*1*J*/*m*1*J*^ controls spending more time on the drug-paired floor than the MAHDR-*Taar1*^+/+^ KI mice. A similar outcome was obtained for the drug-present preference test, with MAHDR-*Taar1*^*m*1*J*/*m*1*J*^ controls spending significantly more time on the drug-paired floor than the MAHDR-*Taar1*^+/+^ KI mice (*p* = 0.007).

Evidence for MA-conditioned preference is indicated by a difference between percent time on the initial test day vs. the two post-conditioning preference test days. For MAHDR-*Taar1*^*m*1*J*/*m*1*J*^ control mice, there was a significant effect of test day [*F*_(2, 94)_ = 21.4, *p* < 0.0001], and *post-hoc* mean comparisons indicated that percent time was greater after MA conditioning when mice were tested under both drug-free and drug-present states (*ps* < 0.001). For MAHDR-*Taar1*^+/+^ KI mice, there was no significant effect of test day; thus, there was no evidence for MA-induced conditioned place preference in these mice.

#### Locomotor Activity During Preference Testing

There was a significant sex × test day interaction [*F*_(2, 184)_ = 3.42, *p* = 0.03]. Activity levels were comparable for males and females during the initial and drug-free preference tests, but males were significantly more active than females during the drug-present test (*p* = 0.03; 3,753 ± 119 and 3,319 ± 157 for males and females, respectively). Sex differences were not dependent on line, but there was a significant line x test day interaction [*F*_(2, 184)_ = 5.49, *p* = 0.005]. Activity levels were comparable between the two genotypes during the initial preference test. Both genotypes increased their activity during the subsequent 2 tests (all *ps* < 0.001), with the highest level of locomotion during the drug-present test and greater activity in MAHDR-*Taar1*^+/+^ KI, compared to MAHDR-*Taar1*^*m*1*J*/*m*1*J*^ control mice (*p* = 0.02 and 0.053 on the drug-free and drug-present test day, respectively).

#### Locomotor Activity During Conditioning

**Figure 3:**
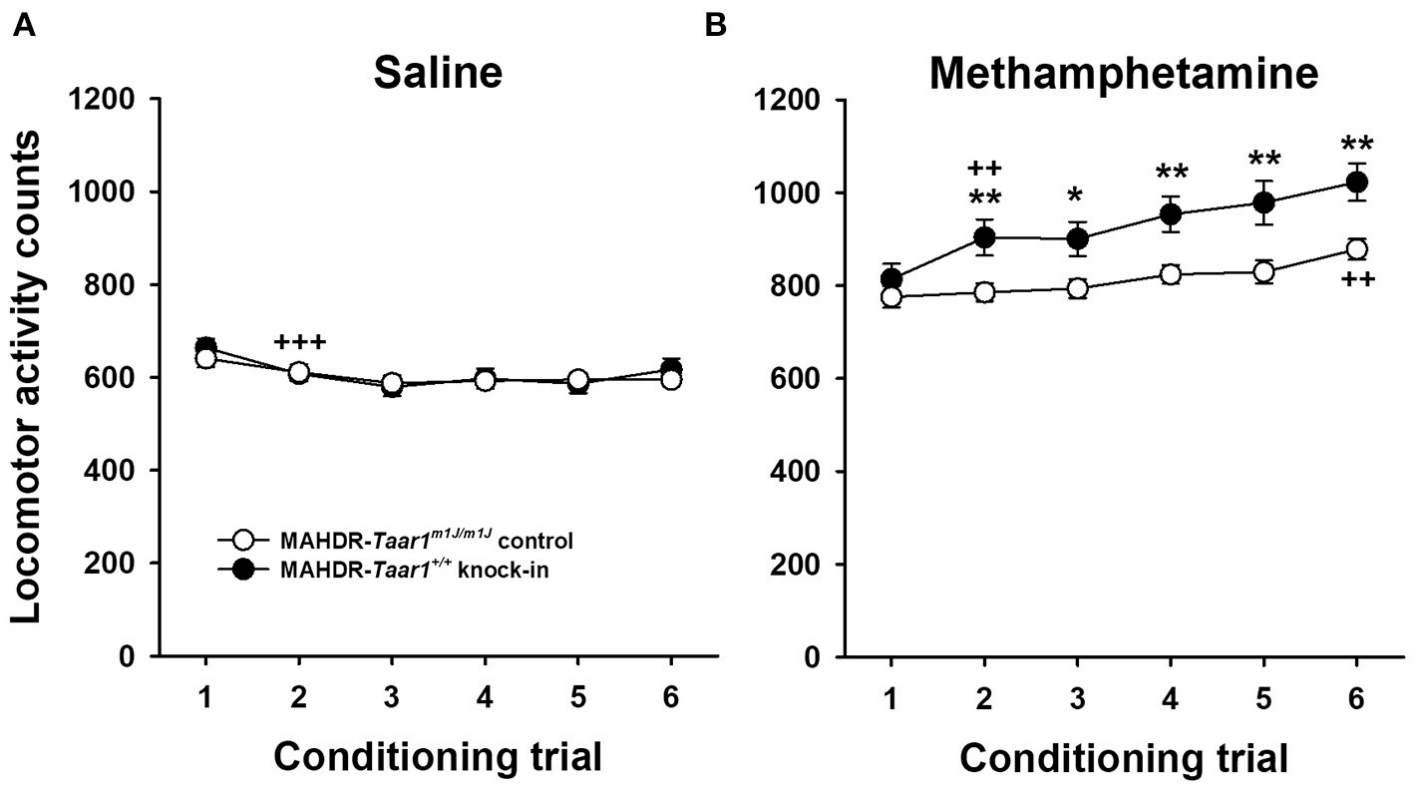
Locomotor activity levels of saline-treated and acute MA-treated MAHDR-*Taar1*^+/+^ KI and MAHDR-*Taar1*^*m*1*J*/*m*1*J*^ control mice are comparable, but locomotor sensitization is more rapid in MAHDR-*Taar1*^+/+^ KI mice. Shown are mean ± SEM locomotor activity counts on each day of conditioning with either **(A)** saline or **(B)** MA. Mice were treated with saline or 0.5 mg/kg MA immediately prior to each 15-min conditioning trial. Mice were the same animals that generated the data in Figure 2. *p < 0.05, **p < 0.01 for the line difference; ^++^*p* < 0.01, ^+++^*p* < 0.001 for the difference from the previous conditioning trial. MA, methamphetamine; MAHDR, methamphetamine high drinking mice; *Taar1*^+/+^, homozygous reference trace amine-associated receptor 1 genotype; *Taar1*^*m*1*J*/*m*1*J*^, homozygous mutant trace amine-associated receptor 1 genotype.

Locomotor activity level data during saline and MA conditioning trials were analyzed for sex, line, and conditioning trial effects. For saline trial data (Figure 3A), there were significant effects of sex [*F*_(1, 92)_ = 7.75, *p* = 0.007] and trial [*F*_(5, 460)_ = 10.58, *p* < 0.0001]. Males were more active than females and activity levels declined significantly from trial 1 to 2 (*p* < 0.001), and were then stable. For MA trial data (Figure 3B), there were no significant sex effects, but there was a significant line × trial interaction [*F*_(5, 460)_ = 2.57, *p* = 0.026]. The mouse lines had comparable activity levels after the first MA treatment, then MAHDR-*Taar1*^+/+^ KI mice were more active than MAHDR-*Taar1*^*m*1*J*/*m*1*J*^ control mice on subsequent trials. Although there was a significant effect of trial within each line (*ps* < 0.001), significant sensitization to the locomotor stimulant effect of MA was found after fewer treatments in MAHDR-*Taar1*^+/+^ KI mice.

### Experiment 3: MA-Induced Conditioned Taste Aversion Testing in MAHDR-*Taar1^+/+^* KI and MAHDR-*Taar1^*m*1*J*/*m*1*J*^* Control Mice

**Figure 4:**
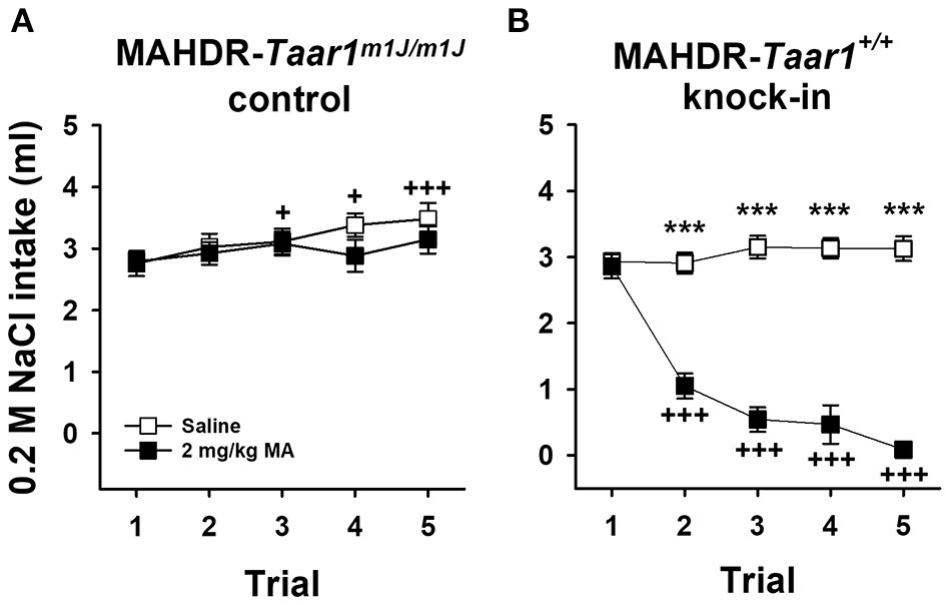
MAHDR-*Taar1*^+/+^ KI mice, but not MAHDR-*Taar1*^*m*1*J*/*m*1*J*^ control mice, exhibit MA-induced conditioned taste aversion. Shown are means ± SEM for 0.2 M NaCl intake in **(A)** MAHDR-*Taar1*^*m*1*J*/*m*1*J*^ control mice and **(B)** MAHDR-*Taar1*^+/+^ KI mice. Consumption trials were separated by 48 h, and saline or 2 mg/kg MA injections (IP) were given immediately after 1 h NaCl access for trials 1–4. Total *N* = 48 mice (6 per line per sex per treatment). ***p < 0.001 for the difference between treatment groups; ^+^*p* < 0.05, ^+++^*p* < 0.001 for the difference compared to trial 1, collapsed on treatment **(A)** or within the MA treatment group **(B)**. MA, methamphetamine; MAHDR, methamphetamine high drinking mice; NaCl, sodium chloride; *Taar1*^+/+^, homozygous reference trace amine-associated receptor 1 genotype; *Taar1*^*m*1*J*/*m*1*J*^, homozygous mutant trace amine-associated receptor 1 genotype.

MAHDR-*Taar1*^+/+^ KI mice, but not MAHDR-*Taar1*^*m*1*J*/*m*1*J*^ control mice, exhibited sensitivity to MA-induced conditioned taste aversion (Figure 4). NaCl intake data were analyzed by repeated measures factorial ANOVA grouped on mouse line, sex, and treatment (saline or 2 mg/kg MA), with test trial as the repeated measure. There was a significant three-way interaction of line, treatment, and trial [*F*_(4, 176)_ = 14.73, *p* < 0.0001], but no significant effect of sex. For the MAHDR-*Taar1*^*m*1*J*/*m*1*J*^ control mice, there was a significant effect of trial [*F*_(4, 88)_ = 4.76, *p* = 0.002], but no effect of treatment; rather than a conditioned reduction in NaCl intake, these mice consumed significantly more NaCl during trials 3–5, compared to trial 1 (*p* = 0.04, 0.04, and 0.0008, for trials 3, 4, and 5, respectively; Figure 4A). For the MAHDR-*Taar1*^+/+^ KI mice, there was a significant trial × treatment interaction [*F*_(4, 88)_ = 34.4, *p* < 0.0001]; there was no significant effect of trial for the saline treatment group, but there was for the MA treatment group (*p* < 0.0001). *Post-hoc* comparisons indicated that NaCl intake was lower for trials 2–5, compared to trial 1 (all *ps* < 0.001; Figure 4B), supporting the development of a conditioned taste aversion in MAHDR-*Taar1*^+/+^ KI mice.

### Experiment 4: Two-Bottle Choice Methamphetamine Intake in DBA/2J*-Taar1^+/+^* KI and DBA/2J*-Taar1^*m*1*J*/*m*1*J*^* Control Mice

**Figure 5:**
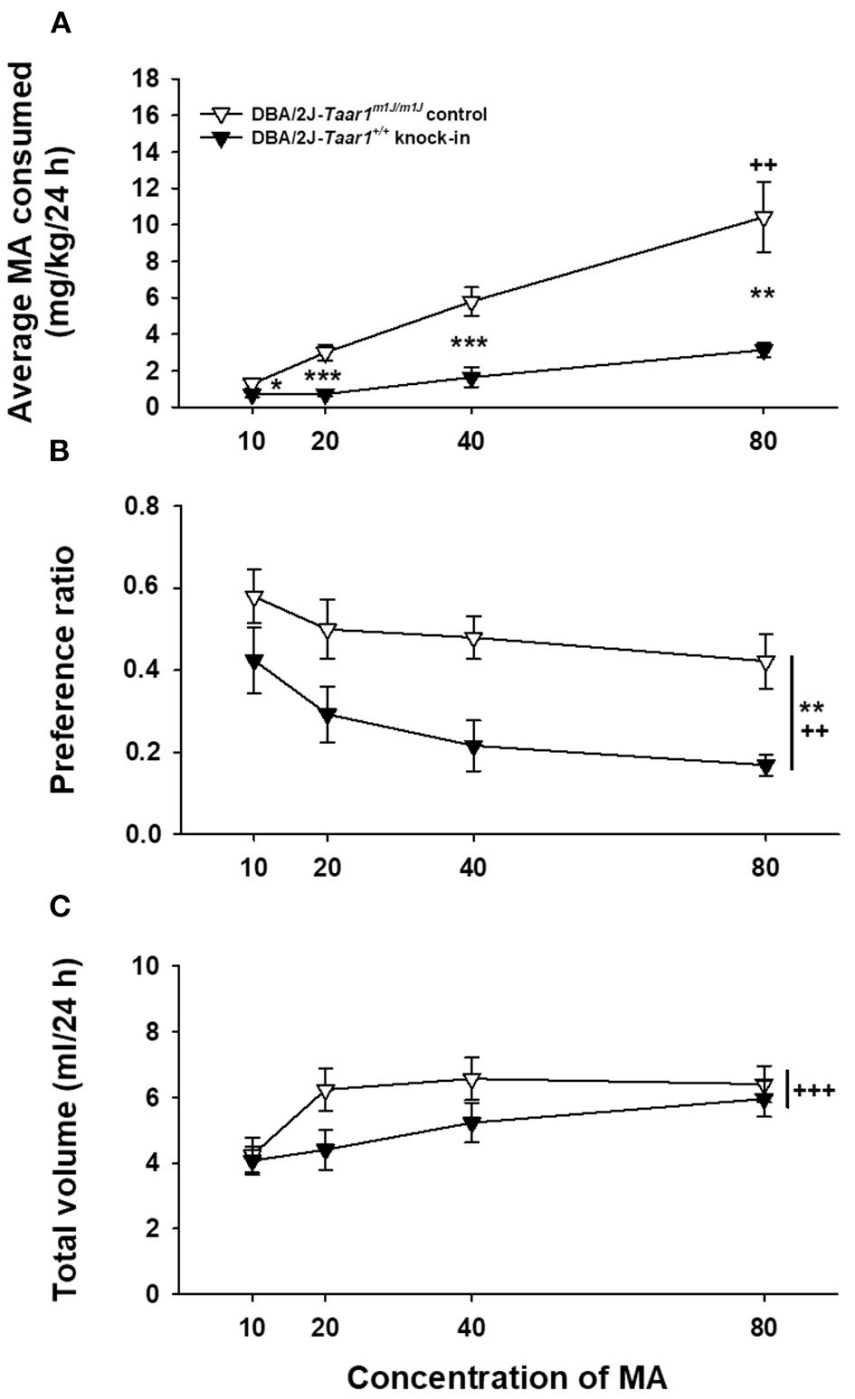
DBA/2J*-Taar1*^*m*1*J*/*m*1*J*^ control mice consume more MA and exhibit greater MA preference, compared to DBA/2J-*Taar1*^+/+^ KI mice. Shown are means ± SEM for **(A)** average MA consumed (mg/kg/24 h), **(B)** preference ratio (ml from MA tube/total ml consumed), and **(C)** total volume consumed (ml/24 h) during two-bottle choice of water and ascending concentrations of MA. Each concentration was offered for a 4-day period. Total *N* = 32 mice (eight per mouse line per sex); *p < 0.05, **p < 0.01, ***p < 0.001 for the difference between mouse lines for a given concentration **(A)** or collapsed on concentration **(B)**; ^++^*p* < 0.01, ^+++^*p* < 0.001 for the effect of concentration compared to the previous concentration **(A)** or for the main effect of concentration **(B,C)**. MA, methamphetamine; *Taar1*^+/+^, homozygous reference trace amine-associated receptor 1 genotype; *Taar1*^*m*1*J*/*m*1*J*^, homozygous mutant trace amine-associated receptor 1 genotype.

DBA/2J*-Taar1*^*m*1*J*/*m*1*J*^ control mice consumed more MA and exhibited greater MA preference, compared to DBA/2J-*Taar1*^+/+^ KI mice (Figure 5). Average MA intake, preference and total volume intake data were analyzed by repeated measures factorial ANOVA grouped on mouse line, sex, and MA concentration. For MA intake (Figure 5A), there was a significant line × MA concentration interaction [*F*_(3, 78)_ = 5.9, *p* = 0.001], but no effect of sex. DBA/2J*-Taar1*^*m*1*J*/*m*1*J*^ control mice consumed more MA than DBA/2J-*Taar1*^+/+^ KI mice at all MA concentrations. Intake significantly increased as MA concentration was increased for DBA/2J*-Taar1*^*m*1*J*/*m*1*J*^ control mice (*ps* < 0.0001), with a statistical trend for KI mice (*p* = 0.06); results for mean comparisons are shown in Figure 5A.

For MA preference (Figure 5B), there was a main effect of line [*F*_(1, 78)_ = 12.9, *p* = 0.001], with DBA/2J*-Taar1*^*m*1*J*/*m*1*J*^ control mice exhibiting a greater MA preference ratio, compared to DBA/2J-*Taar1*^+/+^ KI mice. There was also a significant main effect of concentration [*F*_(3, 78)_ = 5.8, *p* = 0.001]; preference declined with increasing concentration. For total volume consumed (Figure 5C), the only significant effect was MA concentration [*F*_(3, 84)_ = 7.7, *p* = 0.0001]; total volume increased with increasing MA concentration.

### Experiment 5: Two-Bottle Choice Methamphetamine Intake in C57BL/6J-*Taar1^*m*1*J*/*m*1*J*^* KI and C57BL/6J-*Taar1^+/+^* Control Mice

**Figure 6:**
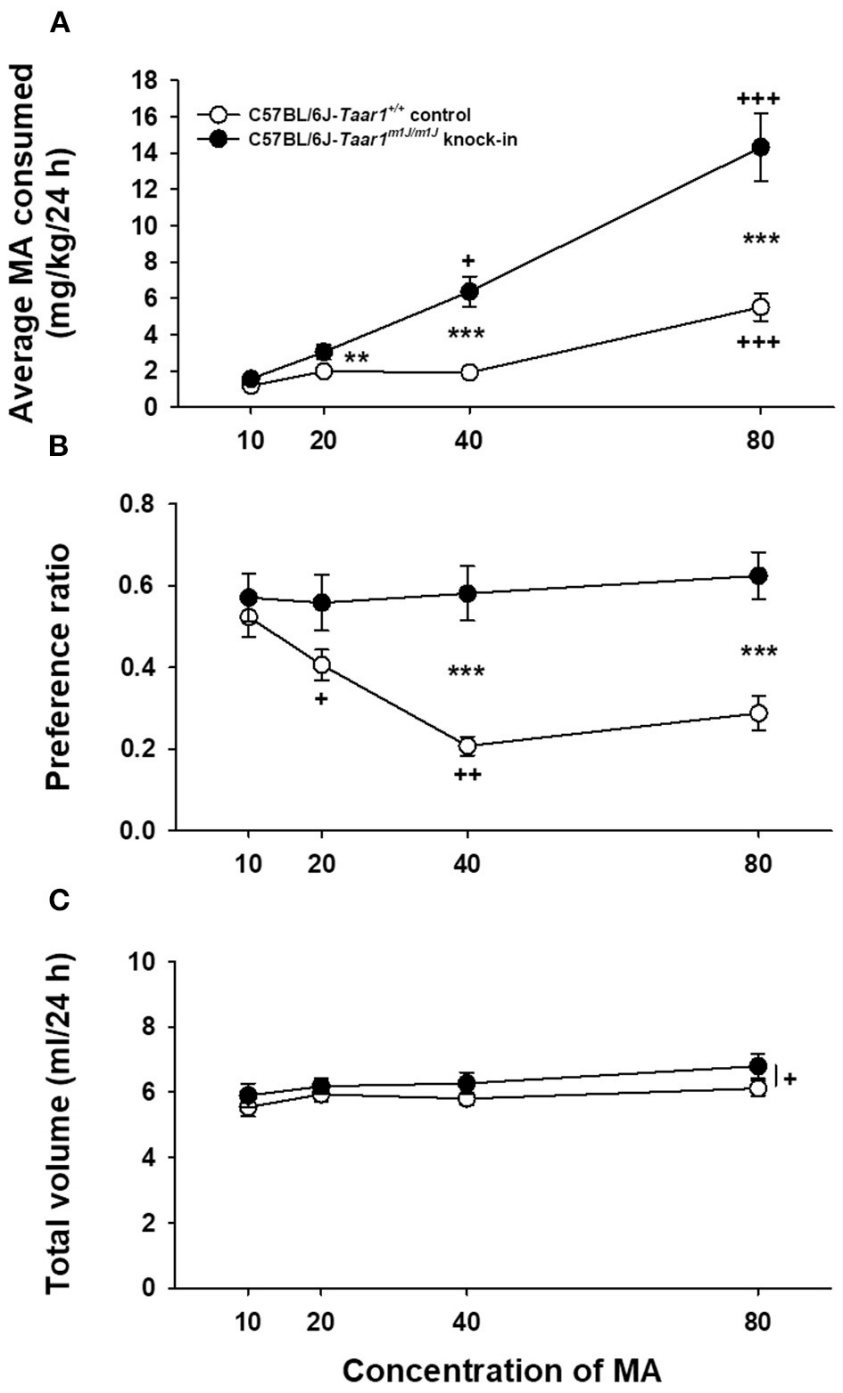
C57BL/6J-*Taar1*^*m*1*J*/*m*1*J*^ KI mice consume more MA and exhibit greater MA preference than C57BL/6J-*Taar1*^+/+^ control mice. Shown are means ± SEM for **(A)** average MA consumed (mg/kg/24 h), **(B)** preference ratio (ml from MA tube/total ml consumed), and **(C)** total volume consumed (ml/24 h) during two-bottle choice of water and ascending concentrations of MA. Each concentration was offered for a 4-day period. Total *N* = 47 (eight mice per sex for the C57BL/6J-*Taar1*^*m*1*J*/*m*1*J*^ KI; 16 male C57BL/6J-*Taar1*^+/+^ control mice, and 15 female C57BL/6J-*Taar1*^+/+^ control mice. **p < 0.01, ***p < 0.001 for the difference between mouse lines; ^+^*p* < 0.05, ^++^*p* < 0.01, ^+++^*p* < 0.001 for the effect of concentration compared to the previous concentration **(A,B)** or for the main effect of concentration **(C)**. MA, methamphetamine; *Taar1*^+/+^, homozygous reference trace amine-associated receptor 1 genotype; *Taar1*^*m*1*J*/*m*1*J*^, homozygous mutant trace amine-associated receptor 1 genotype.

C57BL/6J-*Taar1*^*m*1*J*/*m*1*J*^ KI mice consumed more MA and exhibited greater MA preference than C57BL/6J-*Taar1*^+/+^ control mice (Figure 6). MA intake, preference and total volume intake data were analyzed as described for Experiment 4. For MA intake (Figure 6A), there was a significant line x concentration interaction [*F*_(3, 129)_ = 19.4, *p* < 0.0001]. C57BL/6J-*Taar1*^*m*1*J*/*m*1*J*^ KI mice consumed more MA than C57BL/6J-*Taar1*^+/+^ control mice at all MA concentrations, except 10 mg/L (there was a strong statistical trend, *p* = 0.07). Although there was an increase in intake in both lines across concentration, the increase was steeper in the C57BL/6J-*Taar1*^*m*1*J*/*m*1*J*^ KI mice (see mean comparison results in Figure 6A). There was also a significant line x sex interaction [*F*_(1, 129)_ = 5.4, *p* = 0.025]. However, this interaction was due to a magnitude effect, as there was a significant difference in MA intake between the KI and control mice (*ps* < 0.001) for both males and females (5.2 ± 0.6 mg/kg and 2.7 ± 0.4 for KI vs. control males; 7.5 ± 0.6 and 2.5 ± 0.4 for KI vs. control females), but the difference was 1.9 fold in males and 3 fold in females.

For MA preference (Figure 6B), there was a significant line × concentration interaction [*F*_(3, 132)_ = 5.5, *p* = 0.001], with significantly greater MA preference in C57BL/6J-*Taar1*^*m*1*J*/*m*1*J*^ KI mice compared to C57BL/6J-*Taar1*^+/+^ control mice for the 40 and 80 mg/L concentrations (*ps* < 0.001). There was no significant effect of sex. MA preference decreased in the control mice across increasing concentrations (*p* < 0.0001; see Figure 6B for mean comparisons), but remained stable in the KI mice. For total volume consumed (Figure 6C), there was a significant line × sex interaction [*F*_(1, 44)_ = 13.9, *p* = 0.0006], and a significant effect of concentration [*F*_(3, 129)_ = 3.3, *p* = 0.02]. However, there were no significant increases in fluid consumption from one concentration to the next higher concentration. The line × sex interaction was due to significantly more total volume consumed by female C57BL/6J-*Taar1*^*m*1*J*/*m*1*J*^ KI mice, compared to female C57BL/6J-*Taar1*^+/+^ control mice (27.6 ± 1.2 vs. 22.0 ± 0.9, respectively; *p* = 0.0004), but no significant difference between male mice of the KI and control lines (22.7 ± 1.2 vs. 24.7 ± 0.8, respectively; *p* = 0.17).

### Experiment 6: MA-Induced Body Temperature Change in DBA/2J*-Taar1^+/+^* KI and DBA/2J-*Taar1^*m*1*J*/*m*1*J*^* Control Mice

**Figure 7:**
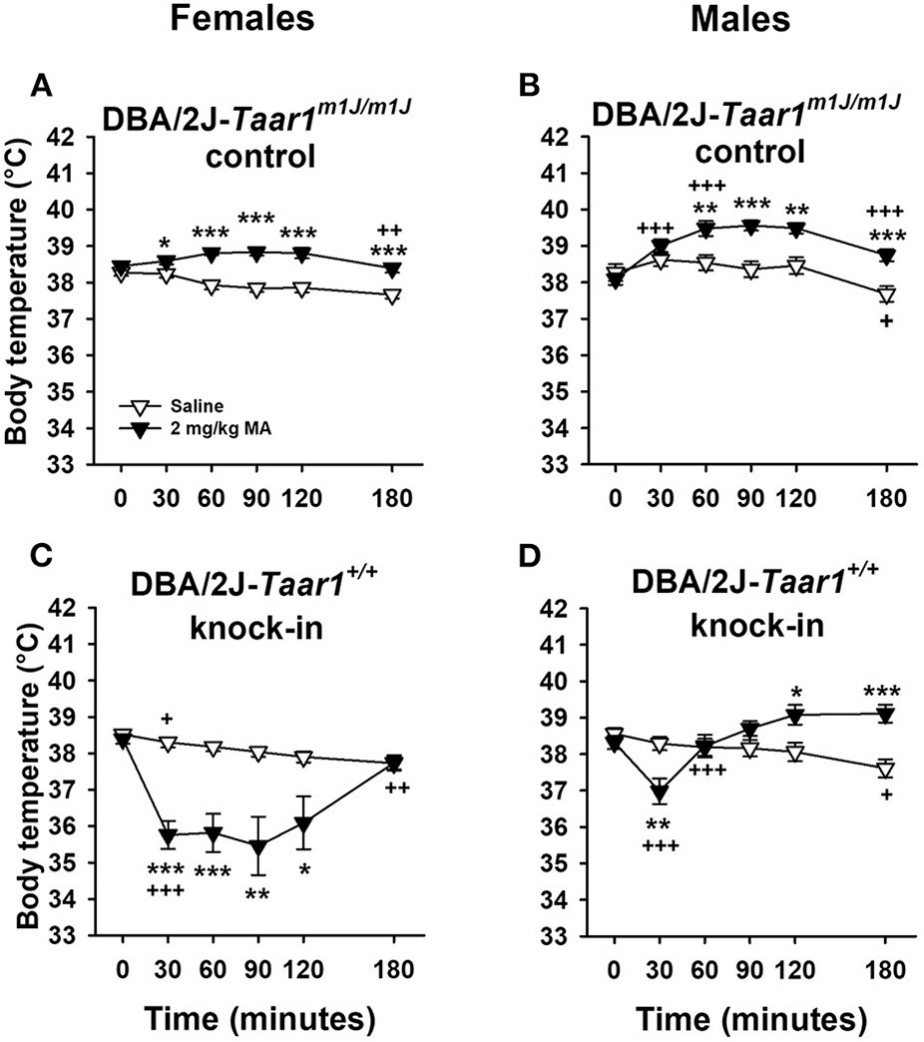
DBA/2J*-Taar1*^+/+^ KI mice display MA-induced hypothermia, whereas DBA/2J-*Taar1*^*m*1*J*/*m*1*J*^ control mice exhibit MA-induced hyperthermia. Shown are means ± SEM for core body temperature (°C) for female **(A)** and male **(B)** DBA/2J-*Taar1*^*m*1*J*/*m*1*J*^ control mice, and for female **(C)** and male **(D)** DBA/2J*-Taar1*^+/+^ KI mice. Data are shown separately for the sexes due to a significant line × sex × treatment interaction. A baseline temperature was obtained (T0), mice were treated IP with saline or 2 mg/kg MA, and then rectal temperatures were obtained periodically from T30 to T180. Total *N* = 81 mice (11 female saline and 10 female MA-treated DBA/2J-*Taar1*^*m*1*J*/*m*1*J*^ control; 10 female DBA/2J*-Taar1*^+/+^ KI per treatment; 10 male per mouse line and treatment). *p < 0.05, **p < 0.01, ***p < 0.001 for the effect of treatment; ^+^*p* < 0.05, ^++^*p* < 0.01, ^+++^*p* < 0.001 for the change in body temperature from the previous temperature. MA, methamphetamine; *Taar1*^+/+^, homozygous reference trace amine-associated receptor 1 genotype; *Taar1*^*m*1*J*/*m*1*J*^, homozygous mutant trace amine-associated receptor 1 genotype.

DBA/2J*-Taar1*^+/+^ KI mice displayed MA-induced hypothermia, whereas DBA/2J-*Taar1*^*m*1*J*/*m*1*J*^ control mice exhibited MA-induced hyperthermia (Figure 7). An interaction with sex was associated with a longer duration of the difference in body temperature response between the two lines in females than in males. Thus, the sex difference did not impact the conclusion regarding the impact of the genetic manipulation. The following analyses support our conclusions. Body temperature data were first analyzed by repeated measures factorial ANOVA grouped on line, sex and treatment (saline or 2 mg/kg MA), with time as the repeated measure. There was a significant four-way interaction [*F*_(5, 365)_ = 4.3, *p* = 0.0009]. Because our main interest is in differences between the mouse lines in MA response, we performed ANOVAs to determine if there were effects of line, treatment and time within each sex. In both the males and females, there was a significant line × treatment × time interaction [*F*_(5, 180)_ = 6.4, *p* < 0.0001 for males; *F*_(5, 185)_ = 11.3, *p* < 0.0001 for females]. For both male and female DBA/2J-*Taar1*^*m*1*J*/*m*1*J*^ control mice, there was a treatment × time interaction [*F*_(5, 90)_ = 7.1, *p* < 0.0001 for males; *F*_(5, 95)_ = 9.2, *p* < 0.0001 for females]. Mean differences are indicated in Figures 7A,B. There were no differences in body temperature between the treatment groups at T0. Female DBA/2J-*Taar1*^*m*1*J*/*m*1*J*^ control mice treated with MA had significantly higher body temperatures at T30–T180 than their saline-treated counterparts. A similar difference was found in males, beginning at T60. For both male and female DBA/2J*-Taar1*^+/+^ KI mice, there was a treatment × time interaction [*F*_(5, 90)_ = 18.9, *p* < 0.0001 for males; *F*_(5, 90)_ = 8.8, *p* < 0.0001 for females]. Mean differences are indicated in Figures 7C,D. There were no differences in body temperature between the treatment groups at T0. Female DBA/2J*-Taar1*^+/+^ KI mice exhibited long-lasting hypothermia from T30-T120, whereas males exhibited significant MA-induced hypothermia only at T30. The MA-treated males had significantly higher body temperatures than saline-treated males at T120 and T180.

We next examined the data for line, sex and time differences within each treatment condition. Data are presented in Supplementary Figure 1. For the saline-treated mice, there was a significant effect of time [*F*_(5, 185)_ = 18.9, *p* < 0.0001], but no significant effect of line or sex. Body temperature changes were examined by comparing adjacent means (i.e., change from the prior time point). Body temperature at T180 was significantly lower than at T120 (*p* < 0.0001). For the MA-treated mice, there was a significant 3-way interaction of line, sex and time [*F*_(5, 180)_ = 3.8, *p* = 0.003). There were no differences in body temperature between the lines for the MA-treatment group at T0. Female DBA/2J*-Taar1*^+/+^ KI mice had lower body temperatures than female DBA/2J-*Taar1*^*m*1*J*/*m*1*J*^ control mice after MA treatment at T30–T180. A similar difference was found in males at T30–T90. Significant changes across time are indicated in Supplementary Figure 1.

### Experiment 7: MA-Induced Body Temperature Change in C57BL/6J-*Taar1^*m*1*J*/*m*1*J*^* KI and C57BL/6J-*Taar1^+/+^* Control Mice

**Figure 8:**
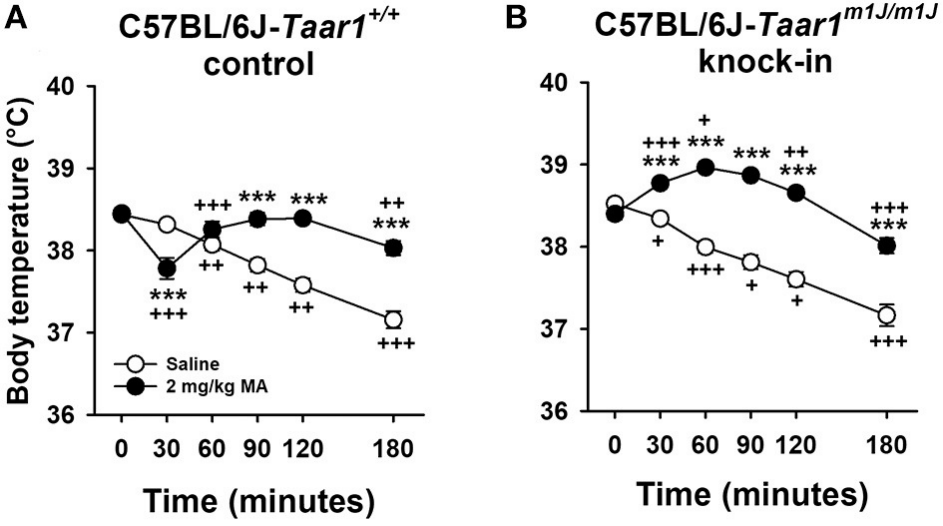
C57BL/6J-*Taar1*^*m*1*J*/*m*1*J*^ KI mice display MA-induced hyperthermia, whereas C57BL/6J-*Taar1*^+/+^ control mice exhibit MA-induced hypothermia. Shown are means ± SEM for core body temperature (°C) for **(A)** C57BL/6J-*Taar1*^+/+^control and **(B)** C57BL/6J-*Taar1*^*m*1*J*/*m*1*J*^ KI mice. A baseline temperature was obtained (T0), mice were treated IP with saline or 2 mg/kg MA, and then rectal temperatures were obtained periodically from T30 to T180. Total *N* = 98 (11 female and 13 male saline-treated C57BL/6J-*Taar1*^+/+^ control; 11 female and 12 male MA-treated C57BL/6J-*Taar1*^+/+^ control; 13 saline-treated C57BL/6J-*Taar1*^*m*1*J*/*m*1*J*^ KI per sex; 12 female and 13 male MA-treated C57BL/6J-*Taar1*^*m*1*J*/*m*1*J*^ KI). ***p < 0.001 for the effect of treatment; ^+^*p* < 0.05, ^++^*p* < 0.01, ^+++^*p* < 0.001 for the change in body temperature from the previous temperature. MA, methamphetamine; *Taar1*^+/+^, homozygous reference trace amine-associated receptor 1 genotype; *Taar1*^*m*1*J*/*m*1*J*^, homozygous mutant trace amine-associated receptor 1 genotype.

C57BL/6J-*Taar1*^*m*1*J*/*m*1*J*^ KI mice displayed MA-induced hyperthermia, whereas C57BL/6J-*Taar1*^+/+^ control mice exhibited MA-induced hypothermia (Figure 8). The following analyses support this summary. Body temperature data were first analyzed by repeated measures factorial ANOVA grouped on line, sex, and treatment (saline or 2 mg/kg MA), with time as the repeated measure. There were two significant three-way interactions; a line × treatment × time interaction [*F*_(5, 450)_ = 20.0, *p* < 0.0001] and a sex × treatment × time interaction [*F*_(5, 450)_ = 7.0, *p* < 0.0001]. There were no interactions of sex with line, indicating that line differences were not sex-dependent.

To examine the line × treatment × time interaction, data were examined for treatment effects within each line. For the C57BL/6J-*Taar1*^+/+^ control mice (Figure 8A), there was a significant treatment × time interaction [*F*_(5, 225)_ = 34.7, *p* < 0.0001]. MA produced significant hypothermia at T30, and the declining temperatures that occurred in the saline group across time were reduced in the MA-treated mice; thus, they had higher body temperatures at T90–T180. For the C57BL/6J-*Taar1*^*m*1*J*/*m*1*J*^ KI mice (Figure 8B), there was a significant treatment × time interaction [*F*_(5, 245)_ = 37.2, *p* < 0.0001]. MA-treated mice had higher body temperatures than saline-treated mice at all time points except T0.

We next examined the data for line and time differences within each treatment condition. Data are presented in Supplementary Figure 2. For the saline group, there was a significant effect of time [*F*_(5, 240)_ = 140.8, *p* < 0.0001], but no body temperature differences between the mouse lines. For the MA treatment group, there was a significant line × time interaction [*F*_(5, 230)_ = 23.0, *p* < 0.0001], and the mouse lines differed in body temperature at all time points except T0 and T180. Significant changes across time are indicated in Supplementary Figure 2.

### Experiment 8: Morphine-Induced Body Temperature Changes in MAHDR-*Taar1^+/+^* KI and MAHDR-*Taar1^*m*1*J*/*m*1*J*^* Control Mice

**Figure 9:**
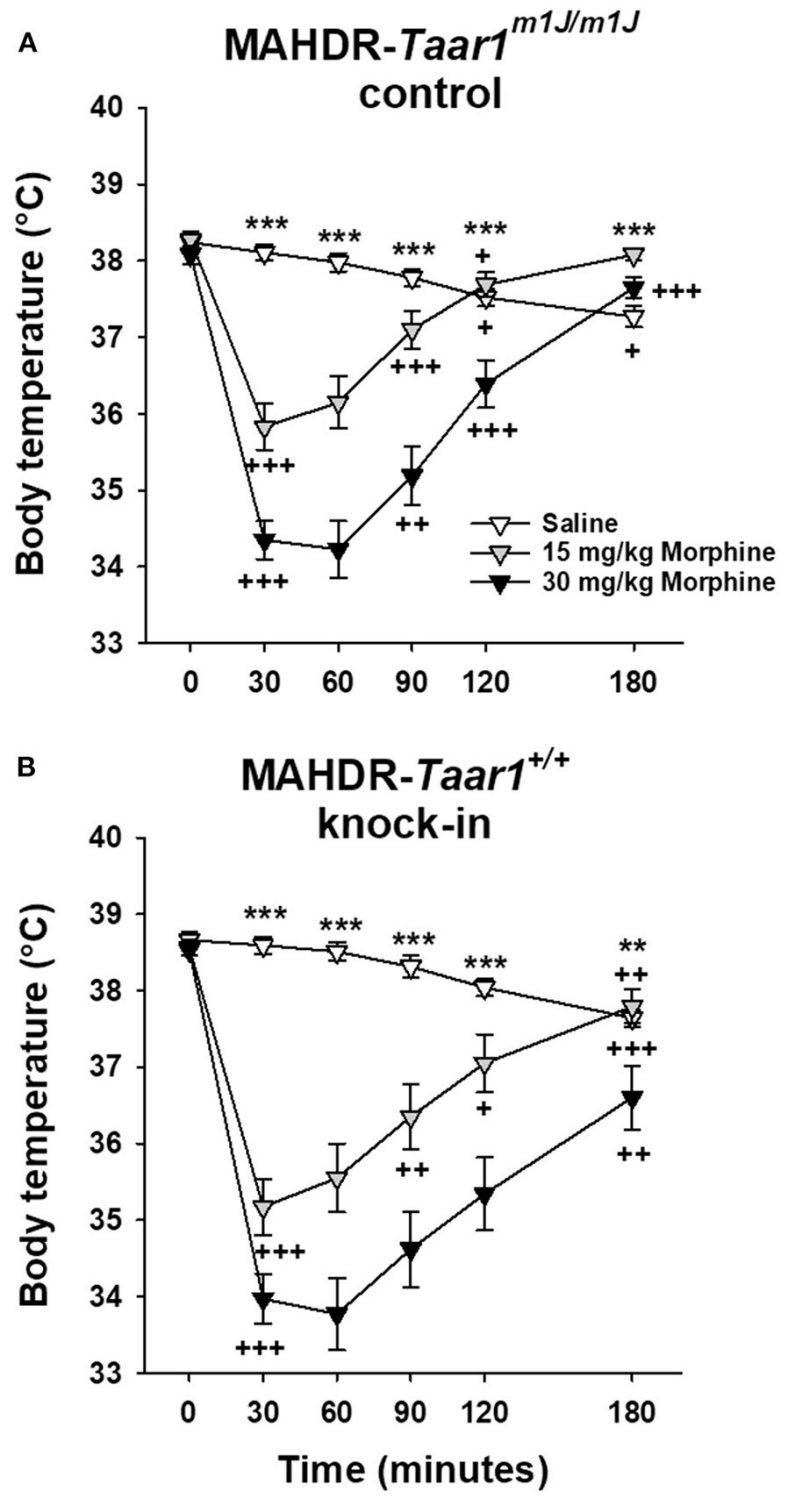
MAHDR-*Taar1*^+/+^ KI and MAHDR-*Taar1*^*m*1*J*/*m*1*J*^ control mice, exhibited comparable sensitivity to morphine-induced hypothermia. Shown are means ± SEM for core body temperature (°C) for **(A)** MAHDR-*Taar1*^*m*1*J*/*m*1*J*^ control and **(B)** MAHDR-*Taar1*^+/+^ KI mice. A baseline temperature was obtained (T0), mice were treated IP with saline, 15 or 30 mg/kg morphine, and then rectal temperatures were obtained periodically from T30 to T180. Total *N* = 140 mice (11 saline-treated MAHDR-*Taar1*^+/+^ KI per sex; 12, 15 mg/kg morphine-treated MAHDR-*Taar1*^+/+^ KI per sex; 11 male and 12 female 30 mg/kg morphine-treated MAHDR-*Taar1*^+/+^ KI; 12 MAHDR-*Taar1*^*m*1*J*/*m*1*J*^ control mice per dose per sex). **p < 0.01; ***p < 0.001 for the effect of treatment; ^+^*p* < 0.05, ^++^*p* < 0.01, ^+++^*p* < 0.001 for the change in body temperature from the previous temperature. MA, methamphetamine; MAHDR, methamphetamine high drinking mice; *Taar1*^+/+^, homozygous reference trace amine-associated receptor 1 genotype; *Taar1*^*m*1*J*/*m*1*J*^, homozygous mutant trace amine-associated receptor 1 genotype.

MAHDR-*Taar1*^+/+^ KI and MAHDR-*Taar1*^*m*1*J*/*m*1*J*^ control mice, exhibited comparable sensitivity to morphine-induced hypothermia (Figure 9). The following statistical outcomes support this conclusion. In the initial 4-way repeated measures ANOVA, the only significant effects involving line were line x morphine dose [*F*_(2, 129)_ = 3.2, *p* = 0.04] and line × time [*F*_(5, 645)_ = 3.6, *p* = 0.003] interactions. However, further examination of the effect of line at each morphine dose, identified no statistically significant differences, and examination of the line × time interaction identified a significant line difference in body temperature only at T0 (*p* < 0.0001), with a higher temperature in MAHDR-*Taar1*^+/+^ KI than MAHDR-*Taar1*^*m*1*J*/*m*1*J*^ control mice of only 0.4°C. Although line did not interact with morphine dose and time, data were analyzed separately for each line to demonstrate that morphine induced significant hypothermia. The outcomes of these analyses are represented in Figures 9A,B. Within each line, there was no effect of dose at T0, but there were significant dose effects at all other time points, supporting morphine-induced hypothermia.

In addition, there was a sex × treatment × time interaction [*F*_(10, 645)_ = 2.2, *p* = 0.02]. When data were examined for sex differences, there was a significant sex × time interaction for the 0 [*F*_(5, 220)_ = 2.6, *p* = 0.03] and 30 mg/kg [*F*_(5, 225)_ = 3.8, *p* = 0.002] dose groups, but not the 15 mg/kg dose group. Further examination of the effect of sex at each time point for the saline treatment dose, identified no statistically significant differences. For the 30 mg/kg dose group, females had lower temperatures than males at T30 (*p* = 0.004) and T60 (*p* = 0.03), reflecting greater morphine-induced hypothermia. The temperature difference was 1.2°C at T30 and 1.3°C at T60; however, this sex effect was not dependent on line and thus, did not impact conclusions regarding the genetic manipulation.

## Discussion

Our research provides new and conclusive evidence indicating that a *Taar1* SNP with a key role in MA intake also impacts sensitivity to MA-induced conditioned place preference, conditioned taste aversion and hypothermia. Furthermore, we demonstrate impacts of *Taar1* genotype on multiple genetic backgrounds and significant reciprocal effects of allele exchange by CRISPR-*Cas9*. Low MA intake is associated with low sensitivity to MA reward and high sensitivity to aversive effects of MA. Furthermore, we confirm prior evidence indicating that a gene in linkage disequilibrium with *Taar1* is responsible for a difference in sensitivity to morphine-induced hypothermia in the MADR mouse lines. Table 2 summarizes our key findings.

**Table 2:** Summary of results for *Taar1* genotype effects for all experiments.

**Exp**	**Trait**	**Background**	**Tastant or Drug, Dose or Conc**.	***Taar1^**+/+**^* vs. *Taar ^***m*1*J*/*m*1*J***^***
(1)	Intake	MAHDR	SACC, 1.6 and 3.2 mM	=
	Preference	MAHDR	SACC, 1.6 and 3.2 mM	=
	Total volume	MAHDR	SACC, 1.6 and 3.2 mM	=
(1)	Intake	MAHDR	QUIN, 0.015 and 0.03 mM	≥
	Preference	MAHDR	QUIN, 0.015 and 0.03 mM	>
	Total volume	MAHDR	QUIN, 0.015 and 0.03 mM	=
(2)	CPP baseline	MAHDR	Saline	=
	CPP drug-free	MAHDR	MA, 0.5 mg/kg then saline test	<
	CPP drug-present	MAHDR	MA, 0.5 mg/kg then 0.5 mg/kg test	<
(3)	CTA	MAHDR	MA, 0 and 2 mg/kg	>
(4)	Intake	DBA/2J	MA, 10–80 mg/L	<
	Preference	DBA/2J	MA, 10–80 mg/L	<
	Total volume	DBA/2J	MA, 10–80 mg/L	=
(5)	Intake	C57BL/6J	MA, 10–80 mg/L	<
	Preference	C57BL/6J	MA, 10–80 mg/L	<
	Total volume	C57BL/6J	MA, 10–80 mg/L	<^*^
(6)	Hypothermia	DBA/2J	MA, 0 and 2 mg/kg	>
	Hyperthermia	DBA/2J	MA, 0 and 2 mg/kg	<
(7)	Hypothermia	C57BL/6J	MA, 0 and 2 mg/kg	>
	Hyperthermia	C57BL/6J	MA, 0 and 2 mg/kg	<
(8)	Hypothermia	MAHDR	Morphine, 0, 15, and 30 mg/kg	=

* *This difference was found in females only; Conc., concentration; CPP, conditioned place preference; CTA, conditioned taste aversion; Exp, experiment; MA, methamphetamine; MAHDR, methamphetamine high drinking line; QUIN, quinine; SACC, saccharin; Taar1^+/+^, homozygous reference trace amine-associated receptor 1 genotype; Taar1^m1J/m1J^, homozygous mutant trace amine-associated receptor 1 genotype*.

### Pleiotropic Effects of *Taar1* on MA-Related Traits in MAHDR Mice

MAHDR mice were selectively bred for high levels of voluntary MA intake using a two-bottle choice water vs. MA solution procedure. Subsequent investigation established that taste does not provide an explanation for the difference in MA intake between the MADR lines (16, 18), the MAHDR mice will voluntarily consume binge-like levels of MA (17), and baseline measures, including locomotor activity, measures of learning and memory, body weight, and body temperature, do not systematically differentiate the lines (26). Furthermore, the lines do not differ in responses to cocaine or alcohol, but they do differ in responses to fentanyl, morphine and amphetamine-like drugs, including MDMA (25, 28–30). The current studies investigated whether the *Taar1* SNP that impacts MA intake also plays a part in the reliable differences in MA-induced conditioned place preference, conditioned taste aversion and hypothermia we have observed between the MADR lines (16, 18, 20, 27). Our results confirm an impact of this polymorphism on all three traits.

MAHDR mice consume less morphine and exhibit greater morphine-induced hypothermia than MALDR mice (25, 28). Both *Taar1* and the μ-opioid receptor gene, *Oprm1*, are on mouse chromosome 10, 17 Mb apart. We speculated that the differences in morphine consumption and hypothermia were associated with linkage disequilibrium and more likely an effect of different *Oprm1* alleles inherited from the DBA/2J and C57BL/6J strains, known to impact morphine preference (31, 32). When we examined *Taar1* and *Oprm1* genotype in MADR line mice tested for morphine-induced hypothermia, there was correspondence of magnitude of hypothermia with *Oprm1*, but not *Taar1*, genotype (25). The current data in the MAHDR KI model are consistent with the conclusion that the *Taar1* SNP is not responsible for the difference in morphine-induced hypothermia between the MADR lines, and that another gene(s) is responsible. We have not tested the MAHDR KI mice for all of the traits previously examined in the MADR lines, but would not expect the KI and control lines to differ for cocaine or alcohol responses. We did observe an unexpected difference in quinine intake in experiment 1, but it was the MA-avoiding line that consumed more quinine, so this result does not provide an explanation for higher consumption of bitter-tasting MA. Future studies will track the reliability of this outcome by repeating the study in all of the KI models.

Finally, the results for locomotor activity in the MAHDR KI and control mice agree with our prior findings in the MADR lines that also did not differ in acute locomotor response to 0.5 mg/kg MA. However, the differences found here between the lines, with *Taar1*^+/+^ mice exhibiting greater locomotor activity on subsequent MA treatment trials than *Taar1*^*m*1*J*/*m*1*J*^ mice, were not found for the MALDR and MAHDR lines (16). Although it is possible that this difference is related to *Taar1* genotype, existing studies indicate that mice without *Taar1* function would be more likely to exhibit the greater stimulant response (see section Other examples of single gene identification for addiction-related traits), opposite to our finding. However, differences in those studies were found for higher doses of MA. It is possible that a genetic background difference played a role in the current outcome. Dose-response studies and studies in the other KI and control lines would benefit interpretation.

### Genetic Background Effects

Previous data support an impact of *Taar1* genotype on MA intake and other MA-related traits in multiple mouse models, derived from the DBA/2J and C57BL/6 strains that served as the progenitors of the MADR lines (22). Because many polymorphisms differentiate these strains, more definitive attribution of a trait difference to the *Taar1* SNP is provided by KI models in which the SNP is specifically manipulated. Thus, we generated multiple CRISPR-*Cas9* KI models to provide conclusive evidence of the impact of the *Taar1* SNP and examine potential genetic background effects. In addition, robust effects were observed when the mutant *Taar1* allele was replaced with the reference allele in MAHDR mice (21), but we did not know whether such robust effects would be observed for the reciprocal manipulation. Results for MA intake and preference aligned with *Taar1* genotype across all of the KI models. Thus, *Taar1*^*m*1*J*/*m*1*J*^ mice of both MAHDR and DBA/2J backgrounds consumed more MA and exhibited greater MA preference than their *Taar1*^+/+^ controls [see (21) and Table 2]. Likewise, results for MA treatment-induced temperature changes were aligned with *Taar1* genotype across all of the KI models, with *Taar1*^+/+^ controls exhibiting hypothermic responses that did not occur in *Taar1*^*m*1*J*/*m*1*J*^ mice [see (20) and Table 2].

Although there were similar general outcomes, there were some qualitative differences in results across the models. Because MA intake data for the DBA/2J and C57BL/6 genetic backgrounds were not collected simultaneously, they could not be subjected to direct comparative analysis, but some observations may be worth noting. The effect of exchanging the reference *Taar1* allele with the mutant allele in C57BL/6J mice was an increase in MA intake that peaked at about 14 mg/kg for the 80 mg/L MA concentration, whereas the DBA/2J mice, which naturally possess the mutant *Taar1* genotype consumed about 10 mg/kg MA at the 80 mg/L concentration. Furthermore, the MA intake of mice possessing the *Taar1*^+/+^ genotype on the C57BL/6J vs. DBA/2J background was 5 mg/kg, compared to 3 mg/kg, for the highest MA concentration. This may indicate that there are other genetic variants promoting MA intake in the C57BL/6J strain even in the presence of the protective *Taar1*^+/+^ genotype. This is supported by somewhat higher MA preference in *Taar1*^+/+^ mice of the C57BL/6J background. However, the lower overall MA preference of mice with the *Taar1*^+/+^ genotype, compared to those with the *Taar1*^*m*1*J*/*m*1*J*^ genotype, was clear on both backgrounds. In previous studies, results were compared under identical conditions for DBA/2J and MAHDR mice for MA intake in a binge drinking procedure and for the effect of binge-level drinking followed by withdrawal on depression-like outcomes. MAHDR mice consumed almost twice as much MA as DBA/2J mice. In addition, MAHDR mice displayed greater depression-like symptoms after withdrawal, which may have been related to their higher MA intake (33). Higher MA intake of the MAHDR mice, compared to the DBA/2J, could be due to the presence of C57BL/6J alleles in the MAHDR mice that are permissive for MA intake.

Another apparent difference found in the current studies was a greater reduction over time in body temperature of saline-treated C57BL/6J mice, compared to DBA/2J mice, during isolate housing. This did not impact our ability to detect MA-induced hypothermia, because that effect tends to be most robust within the first 30 min after administration, but it did clearly demonstrate the ability of MA treatment to inhibit the progressive reduction in body temperature.

Rarely have we found sex differences that interact with line in our previous studies of MA-related traits. A significant line × sex × time interaction was observed in the examination of MA effects on body temperature in the DBA/2J KI and control line study, but not the C57BL/6J study. Examination of the patterns of response in Figures 7, 8 indicate a strong similarity in male DBA/2J mice with the overall outcome for the C57BL/6J mice. However, female DBA-*Taar1*^+/+^ KI mice had a markedly prolonged hypothermic response to the 2 mg/kg dose of MA that is more reminiscent of our previous data in MALDR mice (20) and the MAHDR-*Taar1*^+/+^ KI mice (21), although sex differences were not found in those studies. We have speculated that greater sensitivity to hypothermic drug effects may be protective against further drug intake and drug toxicity, and could serve as a marker for reduced psychostimulant addiction risk (25, 34, 35). Additional data are needed to determine if this is a replicable finding worth pursuing.

### Other Examples of Single Gene Identification for Addiction-Related Traits

The successful identification of single gene effects on complex traits, including addiction-related phenotypes, is increasing. Drug-induced stimulation has been of considerable focus, because feelings of stimulation or euphoria in humans appear to contribute to the potential for escalated use (36). Recent data confirmed *Hnrnph1* as a quantitative trait gene for sensitivity to MA-induced stimulation (37). Similar to the way in which *Taar1* was identified, *Hnrnph1* was first implicated in a quantitative trait locus analysis (38), and then gene editing was used to produce a deletion in the first coding exon of the gene and substantiate its role. Not only did this deletion reduce sensitivity to MA stimulation, it also decreased MA-induced reinforcement, reward and dopamine release (39). It is of interest that MA-induced stimulation also tends to be greater in *Taar1*^*m*1*J*/*m*1*J*^ and *Taar1* knock-out mice, both of which lack TAAR1 function and consume more MA or exhibit greater MA reward and reinforcement (16, 18, 20, 40–43). Another study focused on a region of the cannabinoid-1 receptor gene associated with drug and alcohol addiction (44, 45). Deletion using CRISPR-*Cas9* technology reduced expression of the cannabinoid-1 receptor in the hippocampus and also reduced alcohol intake (46). Thus, in recent years, several addiction-relevant genes have been identified using genetic mapping and rapid deletion and KI techniques.

The *Taar1* SNP is a spontaneously occurring mutation that arose in the JAX DBA/2J mice between 2001 and 2003 (22). Such mutations are not rare. For example, a single base pair deletion arose in intron 3 of the C57BL/6J *Gabra2* gene adjacent to a splice acceptor site that results in global reduction of mRNA and protein level expression, compared to levels found in other inbred mouse strains. When CRISPR-*Cas9* was used to repair the deletion, mRNA and protein levels were restored (47). GABRA2 variation has been implicated in alcoholism and drug abuse in human populations (48–52). It is possible that this gene also plays a role in the high alcohol consumption found in C57BL/6J mice, compared to many other strains [e.g., (53, 54)].

Finally, based on previous data supporting an association of the glutamate receptor subunit gene, ionotropic N-methyl-d-aspartate 3A (*GRIN3A*), with nicotine dependence, 16 SNPs were examined in a Chinese Han population. A single SNP association was identified and gene editing was performed in cultured cells using CRISPR-*Cas9* to demonstrate a regulatory function impacting mRNA and protein expression that could be related to differential susceptibility to nicotine dependence (55). The obvious question arises as to whether human TAAR1 variants impact risk for MA addiction. In the mouse, a key feature of *Taar1* involvement in MA intake appears to be initial sensitivity to adverse effects of MA, such as conditioned aversion and hypothermia. In fact, the MALDR mice bred for low MA intake consume a comparable amount the first time MA is offered, precipitously reducing their intake in the next drinking session, presumably after experiencing negative subjective effects (42, 56). The predictive outcome of negative first experiences with amphetamines have not often been studied in humans, although there are a few laboratory-based studies. The general outcome for acute amphetamine and methylphenidate, the two drugs most studied in healthy non-addicted young adults, have documented variation in ratings of arousal, liking and anxiety. Most report positive mood effects, but some report unpleasant effects, and these outcomes predict subsequent session choices of whether or not to take the drug again [see (36)]. None of this research has examined genetic relationships. A recent study by Loftis et al. (57) identified a synonymous *TAAR1* SNP that was associated with higher MA craving in individuals with active MA dependence and in remission, compared to controls with no history of substance dependence. When examined in cell culture, cells transfected with this variant had 40% higher TAAR1 protein expression, compared to cells transfected with the wild-type allele, but no change in protein function. It would be interesting to test this variant in a rodent model of MA craving.

### Potential Shortcomings and Limitations of the Current Work

MA consumption was measured slightly differently at JAX in the DBA/2J and C57BL/6J KI and control mice, compared to the way in which it was measured in our previous studies of the MADR lines and MAHDR KI and controls at the VAPORHCS. At JAX, mice were given access to MA for 24 h/day vs. the 18 h/day established procedure at the VAPORHCS. The JAX method is procedurally simpler, since bottles do not need to be manipulated during the course of the day, as they do for the 18 h/day procedure. Although this reduces our ability to directly compare MA intake amounts across studies conducted at the two locations, there is no issue with evaluating genotype effects using either procedure, as can be seen here and in our data for the MAHDR KI and control mice (21). In fact, a previous study found that MA intake was lower when offered to MAHDR mice for 24 h/day, compared to 18 h/day, but that the difference in MA intake between MAHDR and MALDR mice remained robust (58).

Our MA-induced conditioned place preference, conditioned taste aversion and hypothermia studies examined the effects of only a single MA dose in each case. The dose used was chosen from previous dose-response studies to reliably produce the effects examined here (16, 18, 20, 27). Furthermore, we have found mice with the *Taar1*^*m*1*J*/*m*1*J*^ genotype to be insensitive to the aversive and hypothermic effects of a wide range of MA doses (20, 27); thus, we do not believe that testing additional doses would change the general outcome of the associations described here. Likewise, mice possessing the *Taar1*^+^ allele have exhibited little to no sensitivity to rewarding or reinforcing effects of MA (16, 42).

Our KI mice are produced by separate breeding pairs from those that produce our control mice; thus, the mice are not littermates. However, that is also the case for all mice that possess each of these genotypes, with the exception of non-inbred crosses. For example, the *Taar1*^*m*1*J*^ mutation is found in homozygous form in DBA/2J mice and in some strains of the C57BL/6J × DBA/2J recombinant inbred (BXD RI) series (21, 22, 24, 25). F2 crosses of these mice result in the 3 possible *Taar1* genotypes: *Taar1*^+/+^, *Taar1*^+/*m*1*J*^, *Taar1*^*m*1*J*/*m*1*J*^. *Taar1* genotype—phenotype correlations for MA intake in F2 mice, raised with mixed *Taar1* genotypes among littermates, are comparable to those for MADR line individuals and BXD RI strains (22).

## Conclusions and Future Directions

The *Taar1* SNP at position 229 accounts for 60% of the genetic variance in MA intake in the selectively bred MADR lines (19, 21). Additional research is underway to identify other genes that impact MA intake, including the identification of relevant gene networks [e.g., (15, 19)]. Variance in MA intake in mice with functional TAAR1 is low, whereas variance in mice lacking TAAR1 function is high (17, 22). Data herein and in our published papers indicate that TAAR1 agonist effects of MA are aversive, and we hypothesize that these effects mask rewarding MA effects, strongly inhibiting MA intake. Greater knowledge about the mechanisms by which TAAR1 agonism induces aversion could be leveraged to identify more efficacious treatments for methamphetamine addiction. Because TAAR1 is located intracellularly, MA must be transported into the cell, for example by the dopamine transporter, to gain access. TAAR1 is localized to distinct cellular compartments and signals through different Gα proteins. Thus, cytoplasmic TAAR1 signals via Gαs and adenylyl cyclase, whereas TAAR1 localized to the endoplasmic reticulum signals via Gα13, stimulating the GTPase, RhoA (59). The involvement of these different mechanisms in different aspects of TAAR1 effects is currently unknown, as is the circuitry underlying TAAR1 agonist-induced aversion. The lateral habenula (LHb) encodes negative prediction errors and punishment signals, and LHb activation results in aversive behaviors (60–62). Further, acute MA induces expression of the immediate early gene, fos (63), and lesions of the LHb increase amphetamine-induced stimulation (64). Based on data indicating that glutamate-mediated synaptic plasticity differentiates the MADR lines (15), and data demonstrating differences between the MADR lines in glutamate responses to MA (65, 66), future studies are planned to examine TAAR1 regulation of glutamate synapses in ventral tegmental area dopamine neurons and dorsal raphe serotonin neurons, arising from LHb afferents (67).

Because mice that lack functional TAAR1 are deficient in the opposing aversion mechanism, they have the capacity to experience MA reward. Individual variability in the strength of the rewarding effect may be responsible for residual variability in MA intake in the MAHDR line. Another source of individual variability is genetic modifiers of the *Taar1*^*m*1*J*^ effect in homozygous individuals. We are examining this in the heterogeneous stock—collaborative cross mice developed by our collaborator, Dr. Robert Hitzemann, at the VAPORHCS, which are the product of an 8-way cross of mouse strains representing 89% of the genetic variability present in mice (68, 69). We recently reported the successful selective breeding of mice for higher and lower amounts of MA intake from a population of individuals that are all homozygous for the *Taar1*^*m*1*J*^ allele (70). These lines will allow us to perform transcriptome analyses to identify genetic differences that result in resistance to the enhancing effect of the homozygous *Taar1*^*m*1*J*^ genotype on MA intake, information that could lead to the identification of a new class of therapeutics.

We previously found that although *Oprm1* is not a quantitative trait gene for MA intake (71), it serves as a hub when added to a network of differentially expressed genes derived from nucleus accumbens, prefrontal cortex and ventral midbrain samples from the MALDR and MAHDR lines (19). We confirmed herein that *Taar1* does not impact sensitivity to morphine-induced hypothermia; rather, *Oprm1* likely underlies differential sensitivity to this morphine effect in the MADR lines. This may also be the case for the differential morphine intake of the MADR lines (28), though we have not yet examined this trait in the KI mice. Buprenorphine reduced MA intake in MAHDR mice without impacting total fluid consumption. Lower doses were effective, but higher doses known to have μ-opioid receptor antagonist effects were ineffective, as was the μ-opioid receptor antagonist, naltrexone (28). Morphine, on the other hand, reduced MA intake, but also total fluid intake (71). This suggests that a partial agonist could serve as a treatment to reduce MA intake. To determine whether *Oprm1* plays a role in the effectiveness of buprenorphine, we intend to test BXD RI strain mice that have the high MA intake *Taar1*^*m*1*J*/*m*1*J*^ genotype, but are homozygous for either the DBA/2J or C57BL/6J *Oprm1* allele. If *Oprm1* allele is irrelevant, than effects on MA intake should be comparable across strains.

Finally, it should be noted that *Taar1* agonists and partial agonists are being explored as therapeutics for MA addiction and other neuropsychiatric conditions (72), and have shown promise in animal models (73–75). Of course, the strategy of increasing TAAR1-mediated activity with direct agonists requires a functional receptor, and thus, is not an approach we have been able to take in our genetic mouse models of absent TAAR1 function. However, we have collected data in mice possessing the *Taar1*^+^ allele, and confirmed that TAAR1-specific agonists have strong aversive effects (Shabani and Phillips, unpublished data). It is possible that TAAR1 agonists reduce MA intake via a substitution mechanism (75), but also possible that agonists activate aversion circuitry that reduces the potency of MA reward. We are not aware of reports directly characterizing the subjective effects of TAAR1 agonists in humans.

## Data Availability Statement

The original contributions presented in the study are included in the article/Supplementary Materials, further inquiries can be directed to the corresponding author/s.

## Ethics Statement

The animal study was reviewed and approved by Institutional Animal Care and Use Committee of the VA Portland Health Care System, Portland, OR or the Institutional Animal Care and Use Committee of The Jackson Laboratory, Bar Harbor, ME.

## Author Contributions

TP: experimental design, development of mouse models, statistical analysis, data interpretation, and manuscript writing and revision. TR, SA, HB, JE, and JM: data acquisition, entry, and verification of accuracy. CR: development of experimental protocols, supervision of technical staff, statistical analysis, figure preparation, drafting of methods and results, and manuscript editing. EC: experimental design, development of mouse models, and manuscript editing. All authors contributed to the article and approved the submitted version.

## Conflict of Interest

The authors declare that the research was conducted in the absence of any commercial or financial relationships that could be construed as a potential conflict of interest.

## Publisher's Note

All claims expressed in this article are solely those of the authors and do not necessarily represent those of their affiliated organizations, or those of the publisher, the editors and the reviewers. Any product that may be evaluated in this article, or claim that may be made by its manufacturer, is not guaranteed or endorsed by the publisher.
