# Supplementary material for: Confirmation of a Causal Taar1 Allelic Variant in Addiction-Relevant Methamphetamine Behaviors
Source: Front Psychiatry. 2021 Aug 26;12:725839. doi: 10.3389/fpsyt.2021.725839 (PMC8428522; doi:10.3389/fpsyt.2021.725839)
Supplement: Supplementary file 1 [file Data_Sheet_1.pdf]

Supplementary Figures

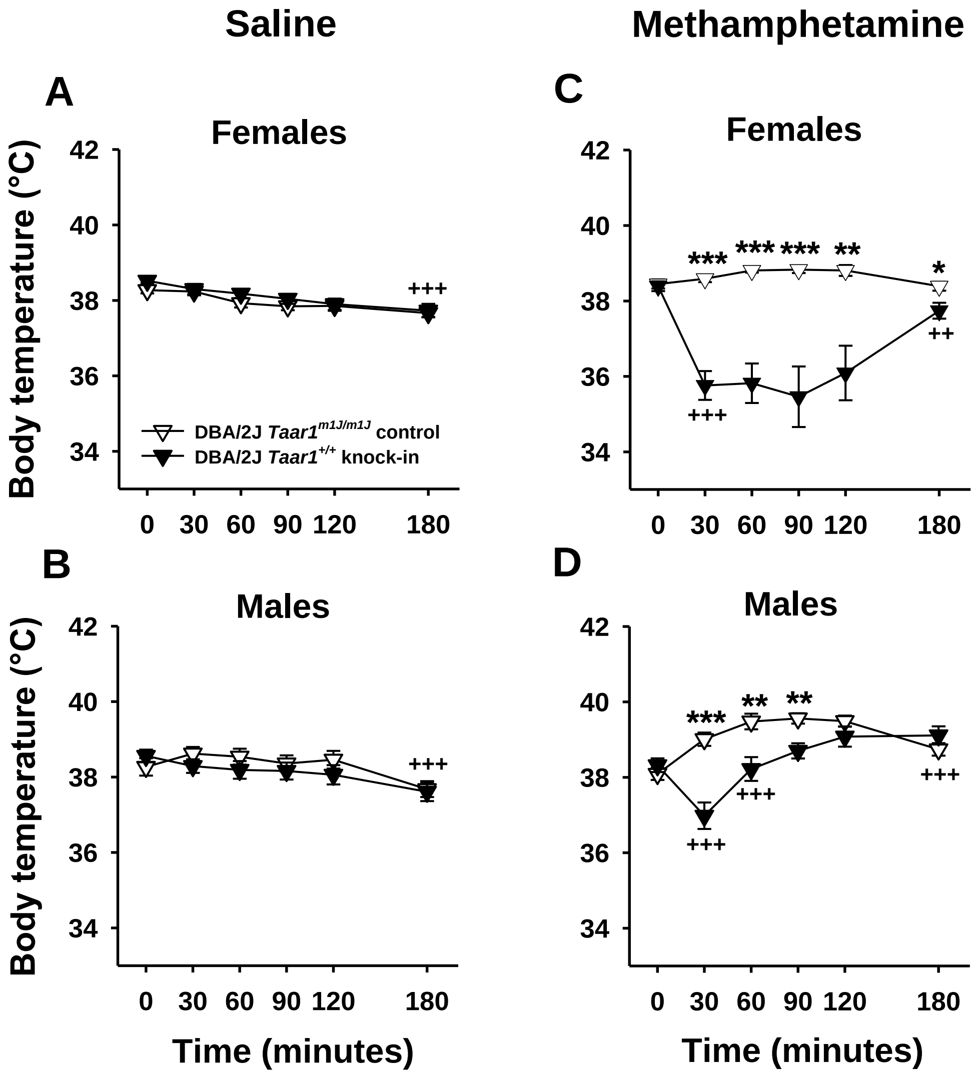

Supplementary Figure 1. Shown are means ± SEM for core body temperature (°C) for (**A**) female and (**B**) male saline-treated DBA/2J-*Taar1*$^{m1J/m1J}$ control and DBA/2J-*Taar1*$^{+/+}$ knock-in mice, and for (**C**) female and (**D**) male methamphetamine-treated DBA/2J-*Taar1*$^{m1J/m1J}$ control and DBA/2J-*Taar1*$^{+/+}$ knock-in mice. Data are shown separately for the sexes due to a significant line x sex x treatment interaction, and illustrate line differences in saline vs. MA responses. *p<0.05, **p<0.01, ***p<0.001 for the effect of mouse line; ++p<0.01, +++p<0.001 for the main effect of body temperature (**A**, **B**), or the change in body temperature from the previous temperature (**C, D**).

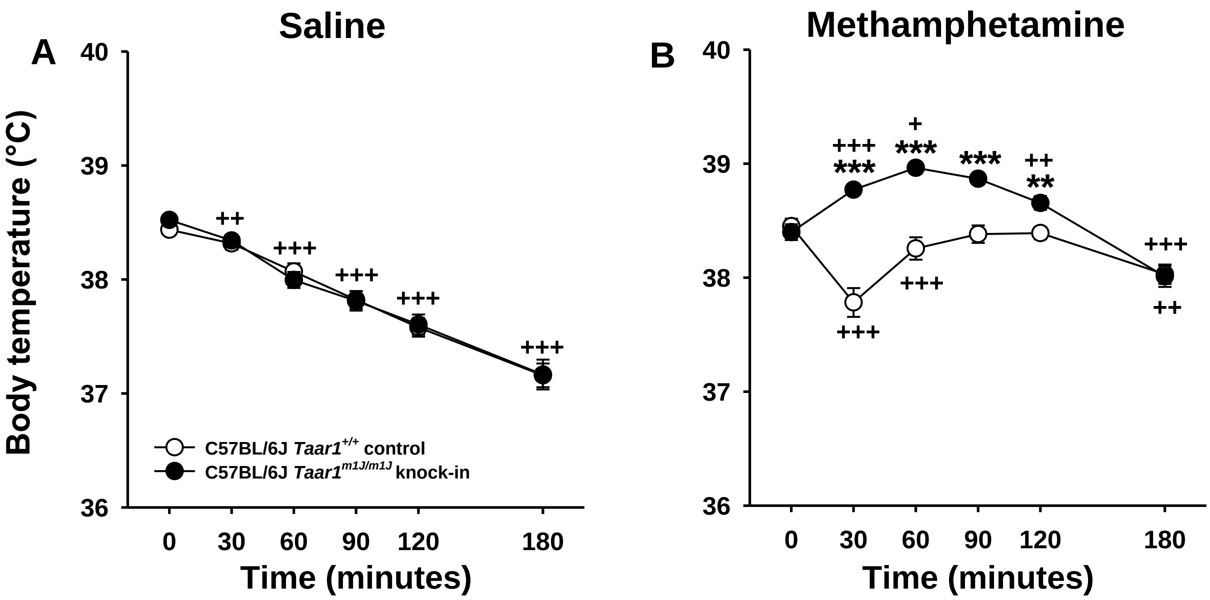

Supplementary Figure 2. Shown are means ± SEM for core body temperature (°C) for (**A**) saline-treated C57BL/6J-*Taar1*$^{+/+}$control and C57BL/6J-*Taar1*$^{m1J/m1J}$ knock-in mice, and (**B**) methamphetamine-treated C57BL/6J-*Taar1*$^{+/+}$control and C57BL/6J-*Taar1*$^{m1J/m1J}$ knock-in mice. This figure illustrates line differences in saline vs. MA responses **p<0.01, ***p<0.001 for the effect of mouse line; +p<0.05, ++ p<0.01, +++p<0.001 for the change in body temperature from the previous temperature, collapsed on line for (**A**).
